# Solution processable diketopyrrolopyrrole (DPP) cored small molecules with BODIPY end groups as novel donors for organic solar cells

**DOI:** 10.3762/bjoc.10.283

**Published:** 2014-11-18

**Authors:** Diego Cortizo-Lacalle, Calvyn T Howells, Upendra K Pandey, Joseph Cameron, Neil J Findlay, Anto Regis Inigo, Tell Tuttle, Peter J Skabara, Ifor D W Samuel

**Affiliations:** 1 WestCHEM, Department of Pure and Applied Chemistry, University of Strathclyde, Glasgow, G1 1XL, UK; 2 Organic Semiconductor Centre, SUPA, School of Physics & Astronomy, University of St. Andrews, St. Andrews, KY16 9SS, UK; 3 Interdisciplinary Centre for Energy Research, Indian Institute of Science, Bangalore 560012, India

**Keywords:** BODIPY, diketopyrrolopyrrole, organic semiconductors, organic solar cells, thiophene

## Abstract

Two novel triads based on a diketopyrrolopyrrole (DPP) central core and two 4,4-difluoro-4-bora-3a,4a-diaza-*s*-indacene (BODIPY) units attached by thiophene rings have been synthesised having high molar extinction coefficients. These triads were characterised and used as donor materials in small molecule, solution processable organic solar cells. Both triads were blended with PC_71_BM as an acceptor in different ratios by wt % and their photovoltaic properties were studied. For both the triads a modest photovoltaic performance was observed, having an efficiency of 0.65%. Moreover, in order to understand the ground and excited state properties and vertical absorption profile of DPP and BODIPY units within the triads, theoretical DFT and TDDFT calculations were performed.

## Introduction

The discovery of photoinduced electron transfer from conjugated polymers to fullerene (C_60_), and the favourable interpenetrating network they form within a bulk heterojunction (BHJ), has led to intense research directed towards the synthesis of conjugated polymers for bulk heterojunction organic photovoltaics (OPVs) [1–3]. In these devices, the conjugated polymer acts as an electron donor and a soluble fullerene, most commonly phenyl-C_61_-butyric acid methyl ester (PCBM), as the electron acceptor [4–9]. However, there is growing interest in the use of small molecules as donor materials in OPVs [10–17]. This interest derives from advantages and properties that small molecules show over conjugated polymers, such as (i) synthetic reproducibility, (ii) higher structural versatility, (iii) ease of purification by recrystallisation and/or chromatography and therefore monodispersity, (iv) higher degrees of crystallinity and (vi) the possibility of vacuum deposition or solution processing during device fabrication. The difference in power conversion efficiencies (PCEs) between polymer and small-molecule based OPVs is decreasing and PCEs over 7% have been realised in the case of the latter [18–19].

**Figure 1:**
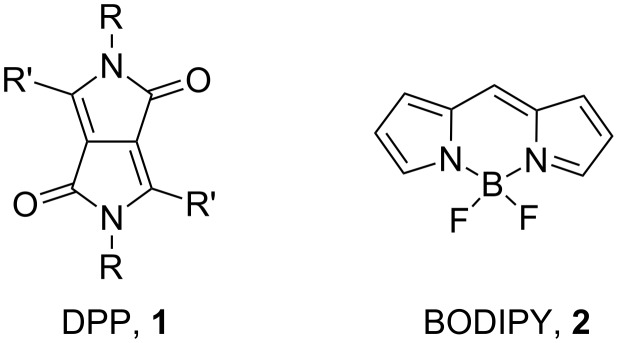
Chemical structures of DPP core **1** and BODIPY core **2**.

Small molecules used in OPVs are most commonly based on oligothiophenes and their derivatives (e.g., selenophene) [20–23], often in combination with other heterocyclic units; the best performing systems are push–pull molecules or dyes [14]. In the last few years the diketopyrrolopyrrole (DPP, **1**, Figure 1) core has been widely incorporated in conjugated polymers for both OPVs and organic field-effect transistors [24–25]. The DPP-based conjugated polymers usually show good electron and hole mobility and promising PCE values in OPVs due to large intermolecular interactions through π–π stacking. Nguyen et al. have investigated the DPP core in small molecules for OPVs with excellent results [26–28]. A PCE greater than 4% was achieved in combination with phenyl-C_71_-butyric acid methyl ester (PC_71_BM) [29]. Interestingly, a small molecule based on a DPP core substituted with electron-withdrawing units was also used as an acceptor in OPVs as a substitute for fullerene with PCEs of 1% when combined with poly(3-hexylthiophene) [30–31].

4,4-Difluoro-4-bora-3a,4a-diaza-*s*-indacene (BODIPY, **2**), and its derivatives have been widely used in the last two decades due to their outstanding chemical and optoelectronic properties [32–34]. BODIPY derivatives are promising compounds to be used in the active layer of OPV devices as they show high absorption coefficients, good photostability and chemical robustness. Although the BODIPY unit has been incorporated in conjugated polymers [35–37] and tested in OPVs with moderate PCEs [38–39], several small molecules containing BODIPY derivatives have demonstrated superior performance in OPVs. Roncali and Ziessel developed a series of small molecules based on BODIPY derivatives by substitution of the fluorine atoms with ethynylglycol chains, achieving PCEs higher than 2% [40–42]. Recently, a new series of BODIPY derivatives grafted with bis-vinylthienyl groups exceeded 4.5% [43].

Although, a few dyads and triads containing both the DPP and BODIPY core have been prepared [44–46], here we present the synthesis and characterisation of two novel BODIPY-DPP-BODIPY triads linked by thiophene bridges. These materials were tested in bulk heterojunction OPVs with moderate power conversion efficiencies.

## Results and Discussion

### Synthesis

**Scheme 1:**
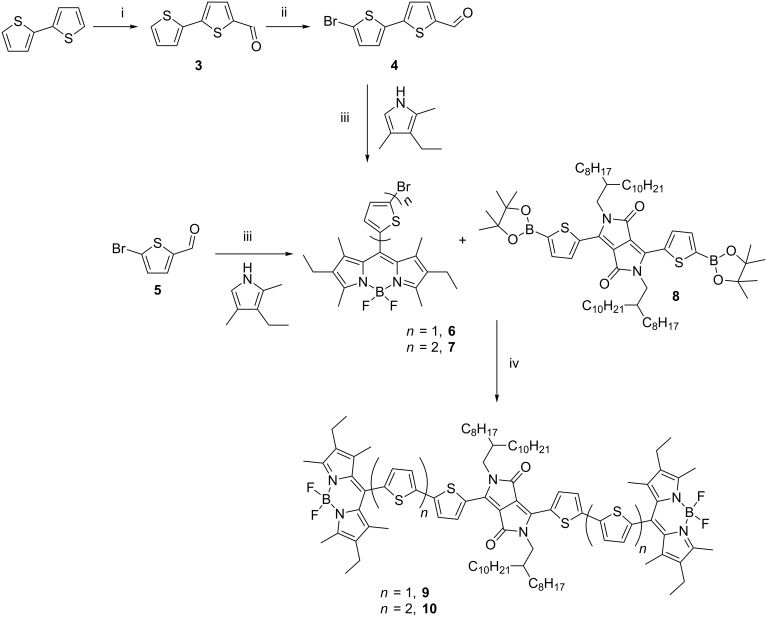
Synthesis of triads **9** and **10**. Reagents and conditions: (i) phosphoryl chloride, *N,N*-dimethylformamide, 50 °C, 16 h, 90%; (ii) NBS, *N,N*-dimethylformamide, rt, 16 h, 88%; (iii) trifluoroacetic acid, dichloromethane, rt, 16 h; DDQ, rt, 24 h; triethylamine, BF_3_·OEt_2_, rt, 24 h, 33% and 12% for **6** and **7**, respectively; (iv) DPP **8**, Pd_2_(dba)_3_, tri-*tert*-butylphosphonium tetrafluoroborate, THF/water, tripotassium phosphate, reflux, 48 h, 55% and 34% for **9** and **10**, respectively.

Our synthetic approach was to prepare BODIPY derivatives bearing a brominated thiophene on the *meso*-position and coupling these derivatives via Suzuki coupling to the central DPP core **8** (Scheme 1). Compound **6** was prepared by acid-catalysed condensation of 5-bromothiophene-2-carbaldehyde with 3-ethyl-2,4-dimethylpyrrole, followed by oxidation with DDQ. Deprotonation with triethylamine and subsequent treatment with boron trifluoride diethyl etherate yielded **6**. The entire synthesis was carried out as a one-pot reaction.

Extending the conjugated π-system of a compound leads to a narrower HOMO–LUMO gap and a bathochromic shift of the absorption spectrum. Both effects are usually desirable to enhance the solar absorption. Therefore, an extended analogue of compound **6** with an additional thiophene ring was synthesised (compound **7**). The synthesis of **7** was achieved from the α-brominated derivative of bithiophene carbaldehyde **4**. This synthetic route to prepare **7** has been described previously in the literature [47]. Formylation of the bithiophene was carried out using Vilsmeier–Haack conditions by treatment with phosphoryl chloride and *N,N*-dimethylformamide to give **3**, which in turn was brominated with NBS (1.05 equiv) to yield compound **4**. Both reactions proceeded in high yields: 90% for the formylation step and 88% for bromination. Compound **7** was prepared following the same procedure used to synthesise **6**. Whereas the synthesis of **6** was achieved in 33% yield, the yield decreased to 12% when the derivative with two thiophenes was prepared [47].

The BODIPY-DPP-BODIPY triads (**9** and **10**) were synthesised via Suzuki–Miyaura cross-coupling by reaction of the functionalised DPP core **8** [48] with the brominated BODIPY derivatives **6** and **7**, respectively. The compounds were purified by standard silica gel column chromatography, but further purification using HPLC was required to isolate compound **10** in sufficiently high purity. Compounds **9** and **10** were thus isolated in 55% and 34% yields, respectively.

### Electrochemical and optical properties

**Figure 2:**
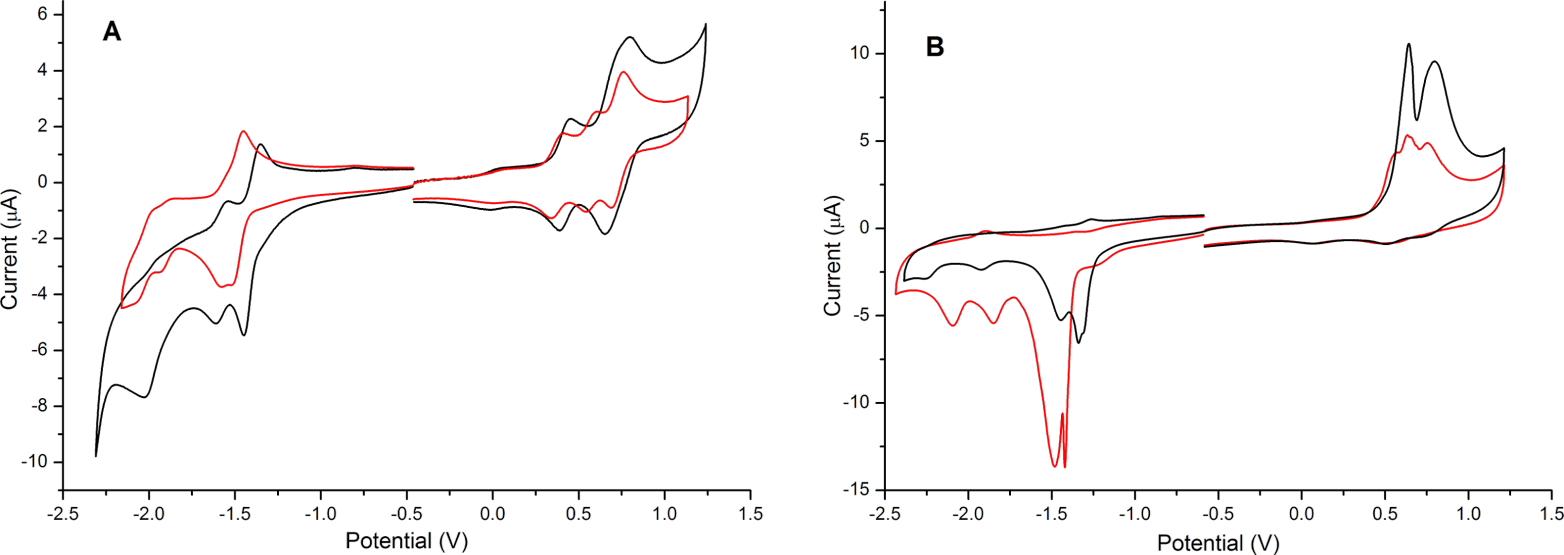
Cyclic voltammetry of **9** (black) and **10** (red) in solution (left) and thin-film (right). The experiments in solution were carried out in dichloromethane (0.1 mM) using a glassy carbon electrode. A film was deposited from a solution of the triads in dichloromethane on a glassy carbon electrode and experiments were carried out in acetonitrile. In both cases, a Ag wire reference electrode and a Pt counter-electrode, in the presence of Bu_4_NPF_6_ (0.1 M), were used. All the values are quoted versus the redox potential of the ferrocene/ferrocenium couple.

The oxidation and reduction processes of **9** and **10** in solution are shown in Figure 2 and Table 1 summarises the corresponding electrochemical data. Upon oxidation, **9** shows two reversible peaks at +0.42 and +0.73 V and **10** shows three reversible processes at +0.37, +0.57 and +0.73 V. In both cases, the first oxidation wave is assigned to the formation of the radical cation on one of the bi/terthiophene segments of the molecule. The lower oxidation potential for **10** compared to **9** is consistent with the tendency to decrease the oxidation potential when the oligothiophene chain is extended. There is then a marked difference in the oxidation behaviour of the two compounds. In **10**, we observe two sequential oxidation processes and ascribe them to the oxidation of the second terthiophene unit, followed by the oxidation of the BODIPY fragment. In **9** these two processes coalesce, albeit at a higher potential.

**Table 1:** Electrochemical data for compounds **9** and **10** in solution and solid state.^a^

Solution state					
	*E*_ox_ [V]	*E*_red_ [V]	HOMO [eV]	LUMO [eV]	HOMO–LUMO gap [eV]

**9**	+0.45/+0.39 +0.80/+0.66	−1.44/1.34^qr^ −1.61/1.54 −2.01^ir^	−5.13	−3.50	1.63

**10**	+0.40/+0.33 +0.60/+0.54 +0.76/+0.69	−1.51/−1.45^qr^ 1.58/−1.52^qr^ −1.93/−1.87^qr^ −2.09^ir^	−5.10	−3.40	1.70

Solid state					
	*E*_ox_ [V]	*E*_red_ [V]	HOMO [eV]	LUMO [eV]	HOMO–LUMO gap [eV]

**9**	+0.64^ir^ +0.79 ^ir^	−1.30^ir^ −1.34^ir^ −1.44^ir^ −1.92^ir^ −2.24^ir^	−5.31	−3.57	1.74

**10**	+0.56^ir^ +0.64^ir^ +0.75^ir^	−1.41^ir^ −1.49^ir^ −1.84^ir^ −2.04^ir^	−5.25	−3.44	1.81

^a^qr represents a quasi-reversible process; ir is an irreversible process.

The reduction processes of the triads are difficult to interpret accurately due to the occurrence of multiple reduction processes. Compound **9** shows two sequential quasi-reversible peaks at −1.39 V and −1.58 V and one irreversible peak at −2.01 V. The reduction behaviour of **10** is more complex with several processes overlapping. Compound **10** displays three quasi-reversible peaks at −1.48, −1.55 and −1.90 V and an irreversible peak at −2.09 V. It is difficult to assign each of these processes accurately, but it is reasonable to assume that the first two reduction processes are due to the reduction of the DPP and BODIPY moieties. By analogy, the reduction waves at higher negative potentials can be due to the reduction of the oligothiophene units, with **10** having a lower reduction potential for the reduction of the thiophenes because of the extended conjugated chain.

The electrochemical study of the triads in the solid state was also analysed. Although a similar pattern of redox processes is observed for oxidation and reduction, the reversibility of these peaks is lost compared to the solution state studies due to the films dissolving in the electrolytic medium in their highly charged states.

The HOMO and LUMO energy levels were calculated from the onset of the first oxidation and reduction waves in both solution and solid state and used to determine the HOMO–LUMO gap. Due to the close interactions between molecules in the solid state, the HOMO–LUMO gap is expected to be lower compared to the HOMO–LUMO gap calculated from the studies in solution. Interestingly though, **9** and **10** show higher HOMO–LUMO gaps in the solid state. The film formation stabilises significantly the HOMO level of both triads (see Table 1), presumably through the interaction of the donor components of the molecules with the corresponding acceptor units via aggregates. Although, the HOMO and LUMO energy levels are lower in the solid state, the stabilisation of the LUMO is not as large as the HOMO and therefore leads to an increase of the HOMO–LUMO gap. Due to the extended conjugated system, it was also expected to obtain a lower HOMO–LUMO energy gap value for **10**. On the contrary, compound **9** displays a slightly lower energy gap both in solution and in the solid state. This difference is unusual for a system with extended conjugation and is addressed later.

**Figure 3:**
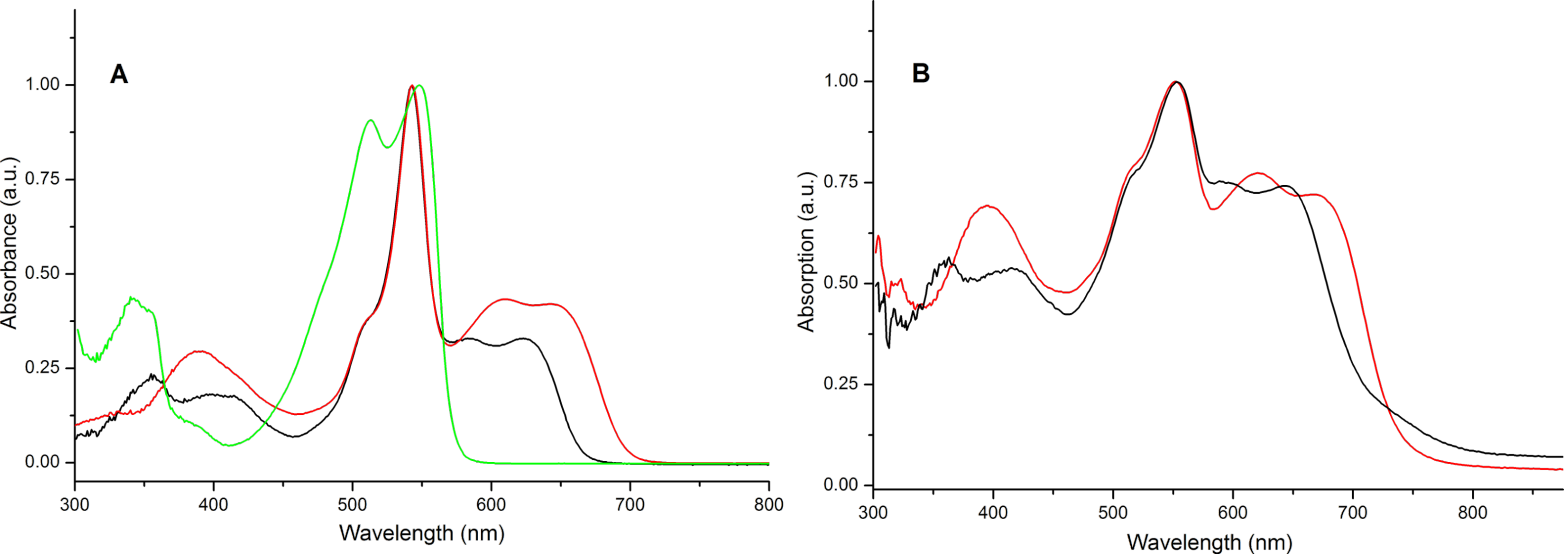
Normalised UV–vis absorption spectra of **9** (black), **10** (red) and DPP core (**11**, green) in dichloromethane solution (left); UV–vis absorption spectra of **9** (black) and **10** (red) core in the solid state, drop-cast from a dichloromethane solution onto ITO (right).

**Figure 4:**
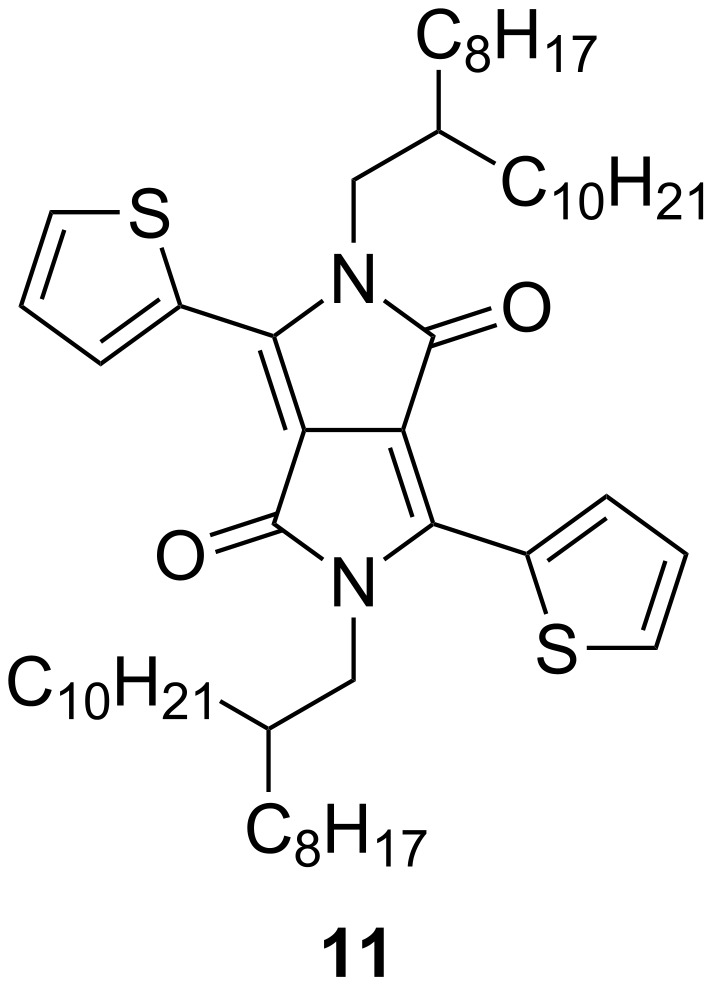
Structure of the dithieno-DPP (**11**) core.

The optical properties of compounds **9** and **10** were characterised by UV–vis absorption spectroscopy in both solution and solid state (drop-cast on ITO) and the corresponding spectra are shown in Figure 3, with the data summarised in Table 2. For comparison, the absorption spectrum of the dithieno-DPP (Figure 4, **11**) core in solution is also shown in Figure 3. All the spectra are normalised to the absorption band for the BODIPY unit. Both triads **9** and **10** show absorption maxima at 542 nm, which is ascribed to the absorption of the BODIPY units. Interestingly, the absorption peak of BODIPY in these compounds is exactly the same value found for a series of BODIPY unit derivatives substituted with oligothiophenes at the *meso*-position [47]. Thus, the incorporation of two extra thiophene rings, and connection to the DPP core, does not affect the absorption peak associated with the BODIPY units. On the other hand, the extension of the π-conjugated system bathochromically shifts the wide absorption band associated with the DPP core and thiophene rings. The DPP core substituted with two thiophenes (compound **11**) showed two intense peaks at 512 and 548 nm. These peaks are red-shifted 72 and 74 nm, respectively, for **9** (584 and 622 nm). The shift is even larger for **10** as the conjugation increases (95 and 97 nm), with compound **10** giving two peaks at 607 and 645 nm. Theoretical calculations support the assignment of these absorption bands to the π–π* transition which is localised on the DPP core (vide infra). Optical HOMO–LUMO gaps in solution were calculated from the onset of the longest wavelength absorption peaks and are summarised in Table 2.

**Table 2:** UV–vis absorption data for compounds **9** and **10** in solution (dichloromethane) and solid state.

	Solution			Solid	
	Absorption peaks [nm]	HOMO–LUMO gap [eV]	*Ε* at 542 nm [dm^3^ mol^−1^ cm^−1^]	Absorption peaks [nm]	HOMO–LUMO gap [eV]

**9**	355, 400 (br), 542, 584, 622	1.86	390,000	423, 554, 591, 643	1.71
**10**	386, 542, 607, 645	1.77	252,000	395, 554, 619, 668	1.67

In the solid state, the triads show the same spectral profile as in solution. In both cases, the main absorption peak occurs at 554 nm. This value is red-shifted 12 nm in comparison with the same peak in solution. The bi/terthiophene-DPP component of the molecule is also red-shifted. A higher degree of order in the solid state is expected compared to the experiments carried out in solution, which shifts both the absorption wavelength and the HOMO–LUMO gaps towards lower energies. The relative intensity of the peak ascribed to the BODIPY unit is diminished compared to the rest of the spectrum. This is due to the higher absorptivity of the thiophene units in the aggregated state. The peaks characteristic of the thieno-DPP sections for **9** and **10** appear at 591 and 643 nm and at 619 and 668 nm, respectively. The HOMO–LUMO gaps of the triads were calculated from the onset of the longest wavelength absorption peaks and are summarised in Table 2. The exact calculation of the HOMO–LUMO gap of **9** is difficult as the onset is diffuse. Nevertheless, the estimated HOMO–LUMO gap of **9** is wider (estimated at 1.71 eV) compared to the energy gap of **10** (1.67 eV) as the extension of the conjugated system leads to a lower HOMO–LUMO gap. Interestingly, whereas in solution the HOMO–LUMO gaps of the triads differ significantly (see Table 2), in the solid state the incorporation of two extra thiophene rings does not decrease the HOMO–LUMO gap to the same extent. The aggregation of **9** in the solid state results in a decrease of the energy gap making it appear similar to **10**, even if the conjugation length is shorter [49].

### Theoretical calculations

**Figure 5:**
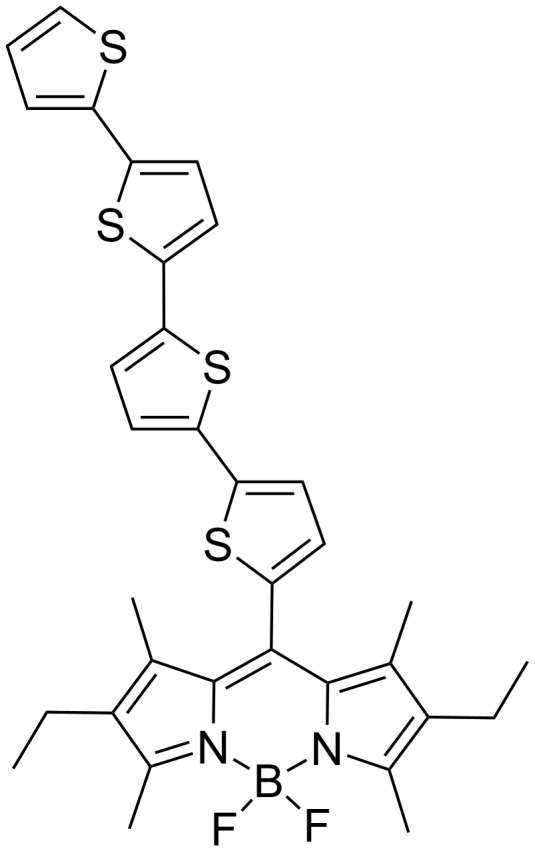
BOD-T4 structure reported by Harriman et al. [50].

DFT optimisations were carried out for compounds **9** and **10**, with the optimised structures for the compounds showing a twist between the BODIPY and thiophene units of 80°, comparable to the 81° twist witnessed in the crystal structure of the BOD-T4 structure (Figure 5) reported by Harriman et al. [50]. The twist of the BODIPY units in these triads suggests that the conjugation extends to the thiophene–DPP central component, isolating the terminal BODIPY moieties.

**Figure 6:**
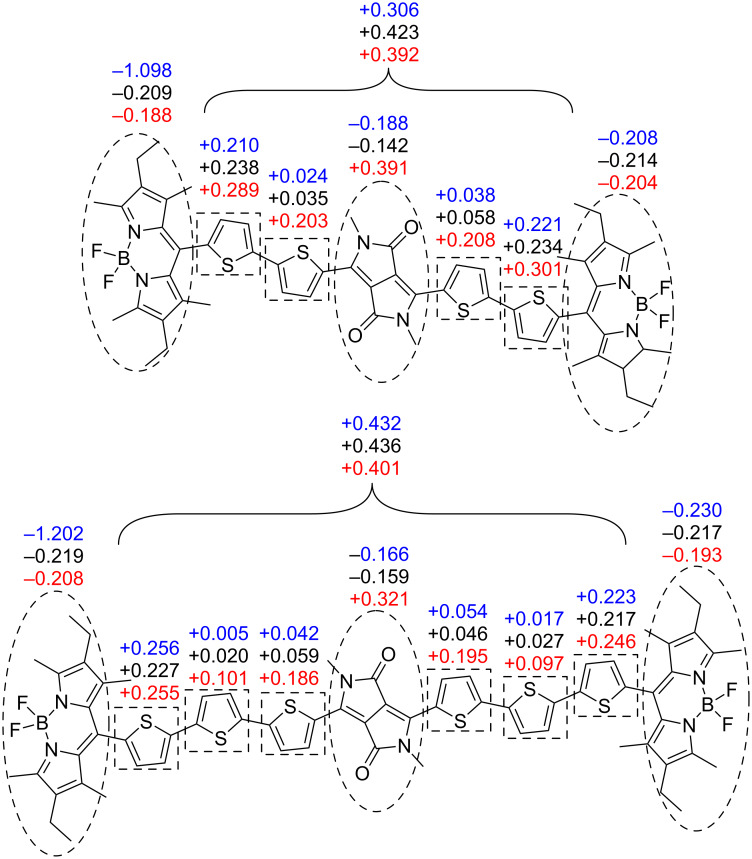
Electrostatic potential charges for each unit in compounds **9** and **10**: radical anion (blue), neutral (black) and radical cation (red) geometries.

**Figure 7:**
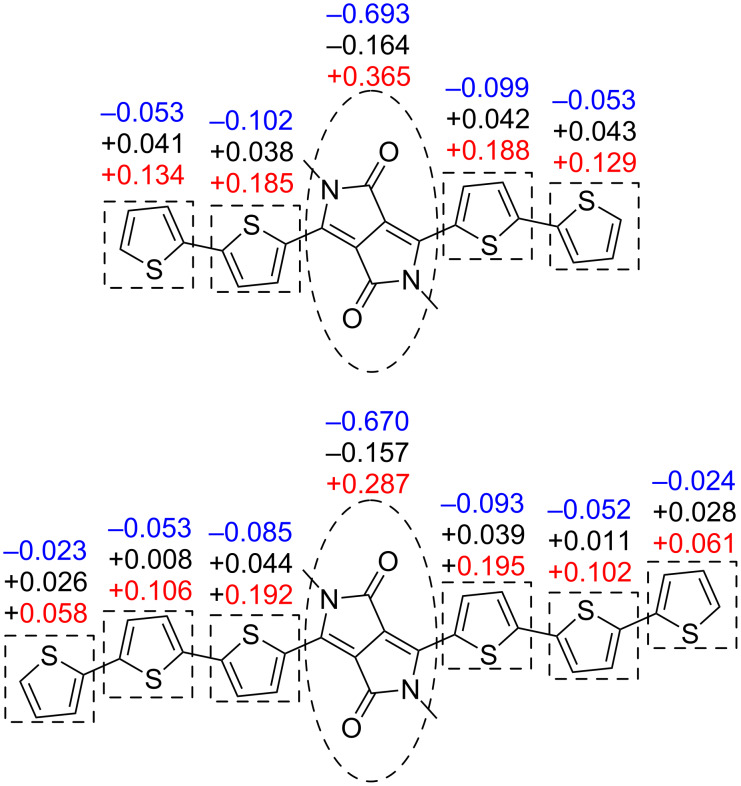
Electrostatic potential charges for each unit in (2Th)_2_DPP and (3Th)_2_DPP radical anion (blue), neutral (black) and radical cation (red) geometries, as analogues of compounds **9** and **10**.

However, despite each BODIPY twisting out of the conjugation plane, these accepting units play an interesting role in the distribution of electrostatic potential charge in the molecules. Shown below are the values of the CHelpG [51] electrostatic potential charge for the component units in compounds **9** and **10** (Figure 6), compared to (2Th)_2_DPP and (3Th)_2_DPP synthesised by Nguyen et al*.* (Figure 7) [27].

The compounds (2Th)_2_DPP and (3Th)_2_DPP in their neutral and radical anion geometries show that the DPP core becomes slightly less negative with increased conjugation, whilst the DPP core becomes less positive with increased conjugation in the radical cation form. The increase in conjugation allows charge to be more evenly distributed across the whole molecule. However, the inclusion of BODIPY accepting units presents a more complex picture. The BODIPY units act as a stronger acceptor than the DPP core, causing the overall electrostatic potential charge of the DPP-oligothiophene unit to be positive. This is observed in all three redox states, with the more conjugated compound **10** more positive in each case. Thus, the electron-accepting ability of the BODIPY units dominate over the effect of increasing the conjugation when comparing compounds **9** and **10** to (2Th)_2_DPP and (3Th)_2_DPP, with an increase in conjugation resulting in more negatively charged BODIPY units.

Using the electrostatic potential charges, along with analysis of the molecular orbitals in compounds **9** and **10**, can help to determine why the reduction wave of the cyclic voltammogram is more negative for compound **10**, despite the increase in conjugation.

**Figure 8:**
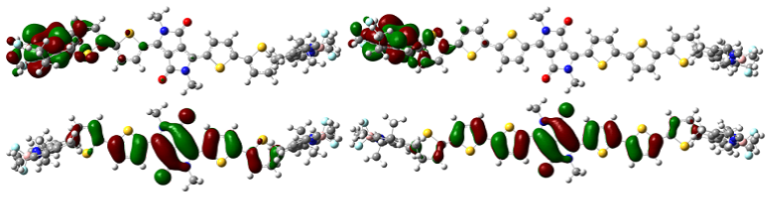
Frontier orbitals for radical anion SOMO (top), neutral HOMO (bottom) of **9** (left) and **10** (right).

The SOMO of the radical anion of each compound is localised on a BODIPY unit (Figure 8), meaning reduction of compounds **9** and **10** should occur at the BODIPY unit. The electrostatic potential calculations in Figure 6 show the BODIPY units in compound **10** to be more negatively charged than in compound **9**. Increased negative charge on the BODIPY would result in a higher energy barrier for reduction, resulting in a greater reduction potential in compound **10**. This in turn contributes to the larger HOMO–LUMO gap determined by cyclic voltammetry for compound **10** with respect to compound **9**.

In addition to the DFT calculations, TDDFT was also carried out in order to investigate the vertical absorption profile of the triads in more depth and the results are shown below in Table 3.

**Table 3:** TDDFT results.

Compound	Calculated absorption peaks [nm]	Transitions

**9**	612	HOMO→LUMO (75%); HOMO−3→LUMO+2 (12%); HOMO−3→LUMO+3 (13%)
	510	HOMO−2→LUMO+1 (32%); HOMO−2→LUMO+2 (12%); HOMO−1→LUMO (9%); HOMO−1→LUMO+1 (15%); HOMO−1→LUMO+2 (32%)
	370	HOMO−4→LUMO (20%); HOMO−4→LUMO+2 (13%); HOMO−3→LUMO+1 (24%); HOMO−3→LUMO+3 (7%); HOMO→LUMO+2 (27%); HOMO→LUMO+4 (9%)
**10**	641	HOMO→LUMO (83%); HOMO−3→LUMO+3 (17%)
	510	HOMO−3→LUMO+1 (35%); HOMO−2→LUMO+1 (23%); HOMO−1→LUMO+2 (42%)
	403	HOMO−4→LUMO (32%); HOMO−3→LUMO+1 (14%); HOMO−3→LUMO+3 (23%); HOMO→LUMO+2 (10%); HOMO→ LUMO+4 (21%)

The lowest energy transition is attributed to the excitation of the thiophene–DPP portion of the molecule (Figures S17 and S20, Supporting Information File 1), whilst the excitation that appears at 510 nm in the TDDFT data for both compounds is the result of absorption by the BODIPY units (Figure S18 and S21, Supporting Information File 1). The difference between the lowest energy peaks in compounds **9** and **10** determined by TDDFT is 29 nm (exp = 23 nm), whilst the BODIPY peaks are in identical positions; this is also evident experimentally. The good agreement between the computational and experimental results shows that wB97XD/TDDFT can be a useful tool in predicting the absorption of BODIPY-based triads and compounds with multiple absorbing units, which are normally difficult to describe computationally.

### Device characterisation

**Figure 9:**
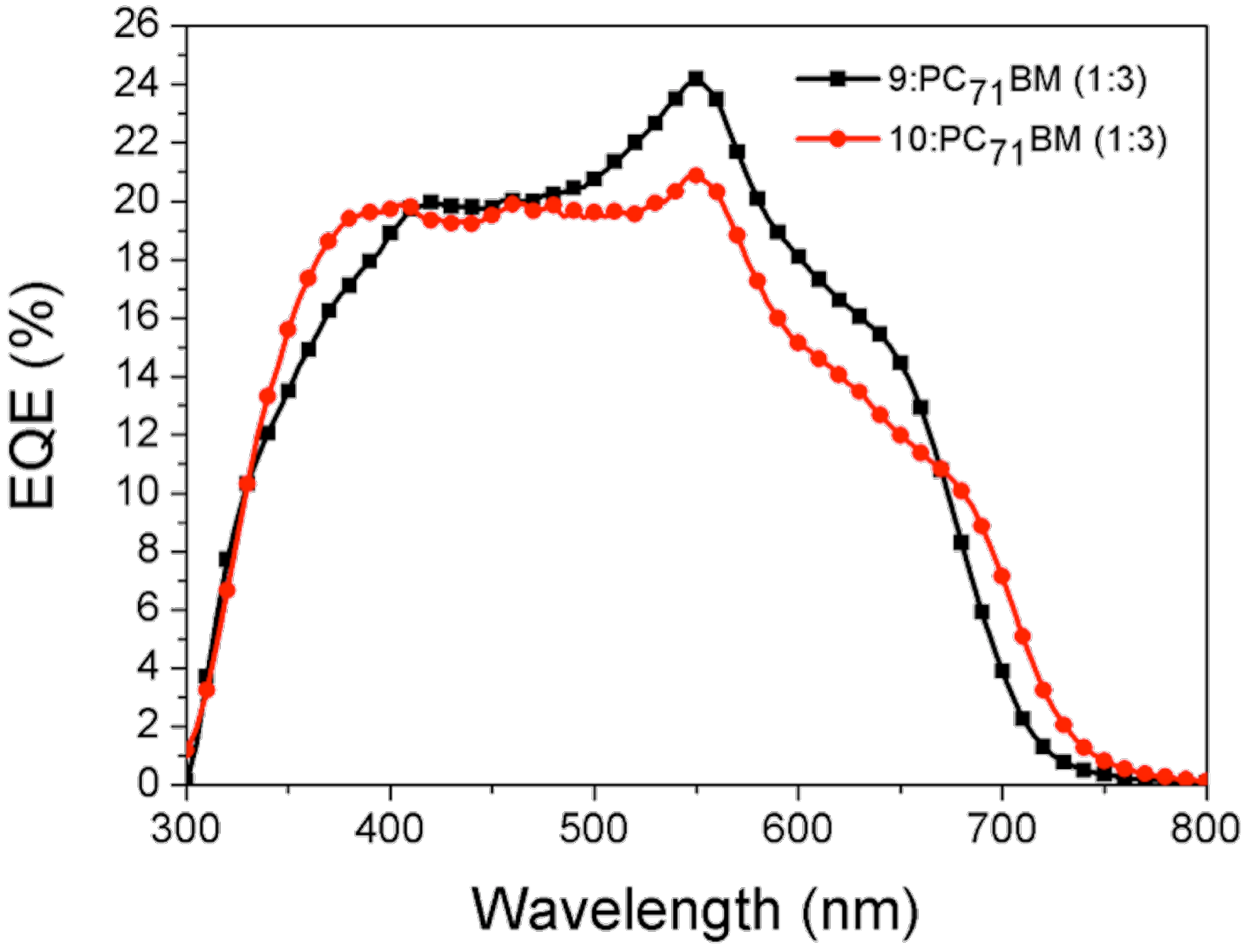
Incident photon to converted electron (IPCE) ratio or external quantum efficiency (EQE) for **9**:PC_71_BM (1:3) (black) and **10**:PC_71_BM (1:3) (red).

Thin films of compound **9** and **10** have favourable absorption when used as a donor for organic photovoltaic applications, absorbing in the region 500–700 nm (Figure S1, Supporting Information File 1). PC_71_BM, which absorbs in the range 300–500 nm, was used as an acceptor with these compounds as it gives favourable energy level matching for efficient devices. Spin-coated films of the donor and acceptor blend allows excellent absorption from 300–700 nm. The absorption spectra of **9**:PC_71_BM and **10**:PC_71_BM with a 1:1 ratio are shown in Figure S2 (Supporting Information File 1). Here we see an enhanced absorption in the region where **9** and **10** have poor absorption (300–500 nm). Absorption is an important process in the function of an organic solar cell but, as previously discussed, it is not the sole process in the operation of an organic solar cell. The dissociation of the coulombically bound electron–hole pair or exciton into free charge and their transport to the electrodes are critical for device operation. Dissociation and transport are two processes that are strongly linked with the morphology of the active layer. Therefore, controlling the morphology can lead to improved device performance. A common technique to optimise morphology is to vary the donor/acceptor ratio. A screening of various donor/acceptor ratios revealed that the most promising performance was evident with the ratio 1:3 (Figures S15 and S16, Supporting Information File 1). As the concentration of the acceptor is increased from 2:1 to 1:3 an increase in short-circuit current (*J*_sc_), open circuit voltage (*V*_oc_) and fill factor (FF) was observed. With further increase in the concentration of the acceptor to 1:4 we observed a decrease in *J*_sc_, *V*_oc_ and FF. Larger concentrations of the acceptor in the active layer enhance absorption in the region 300–500 nm (Figure S3, Supporting Information File 1). This is not ideal for photovoltaic operation as the majority of the solar spectrum is above 500 nm. However, considering the incident photon to current conversion efficiency (IPCE) (Figure 9) of our fully optimised devices (Figures S13 and S14, Supporting Information File 1) we see for both DPP core derivatives an EQE response from 500–700 nm. The EQE spectra indicate that the small molecules are indeed contributing to the overall photocurrent of the devices. Furthermore, the peak at 550 nm is easily identified as the BODIPY core.

**Figure 10:**
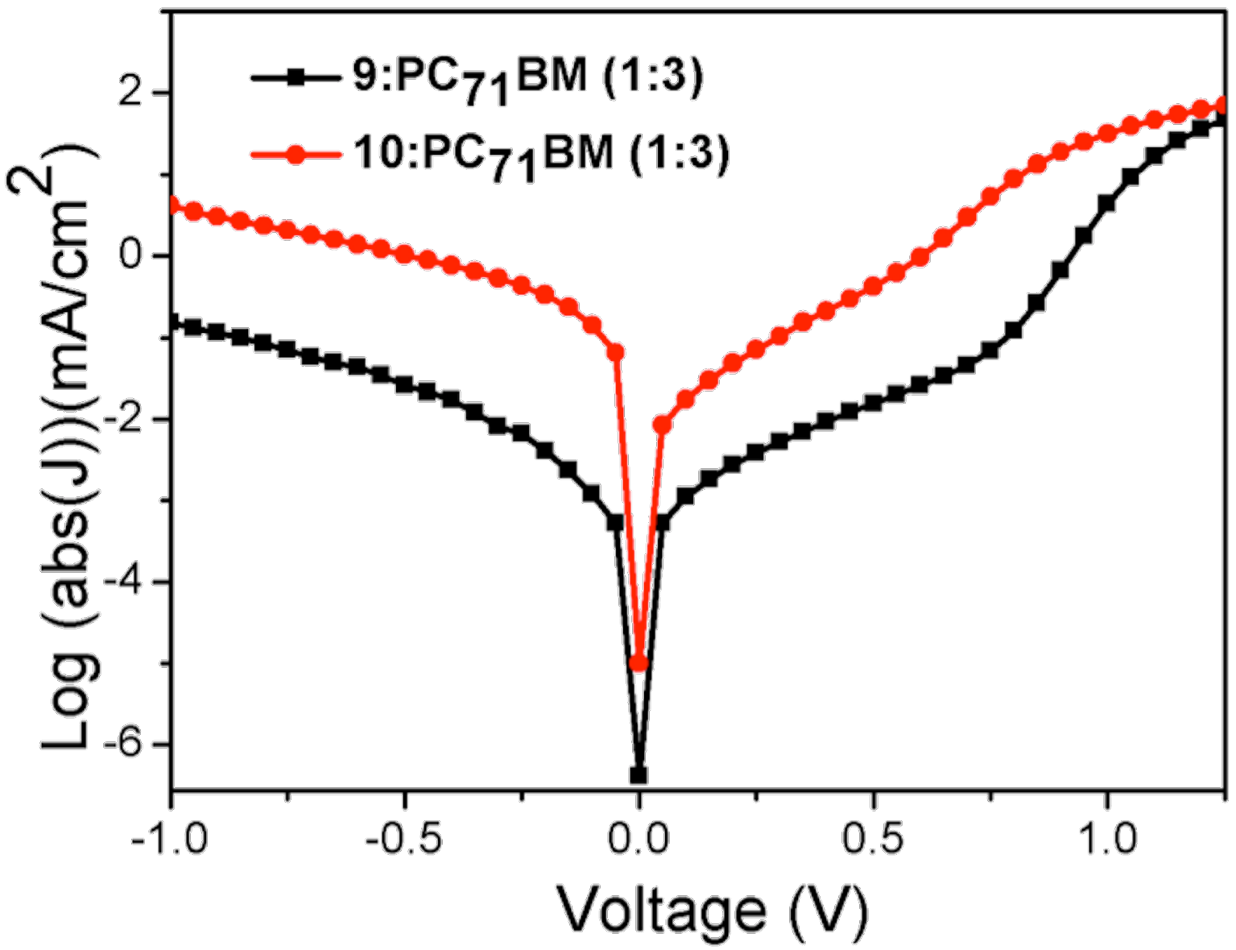
*J*–*V* for **9**:PC_71_BM (1:3) and **10**:PC_71_BM (1:3) in the dark.

**Figure 11:**
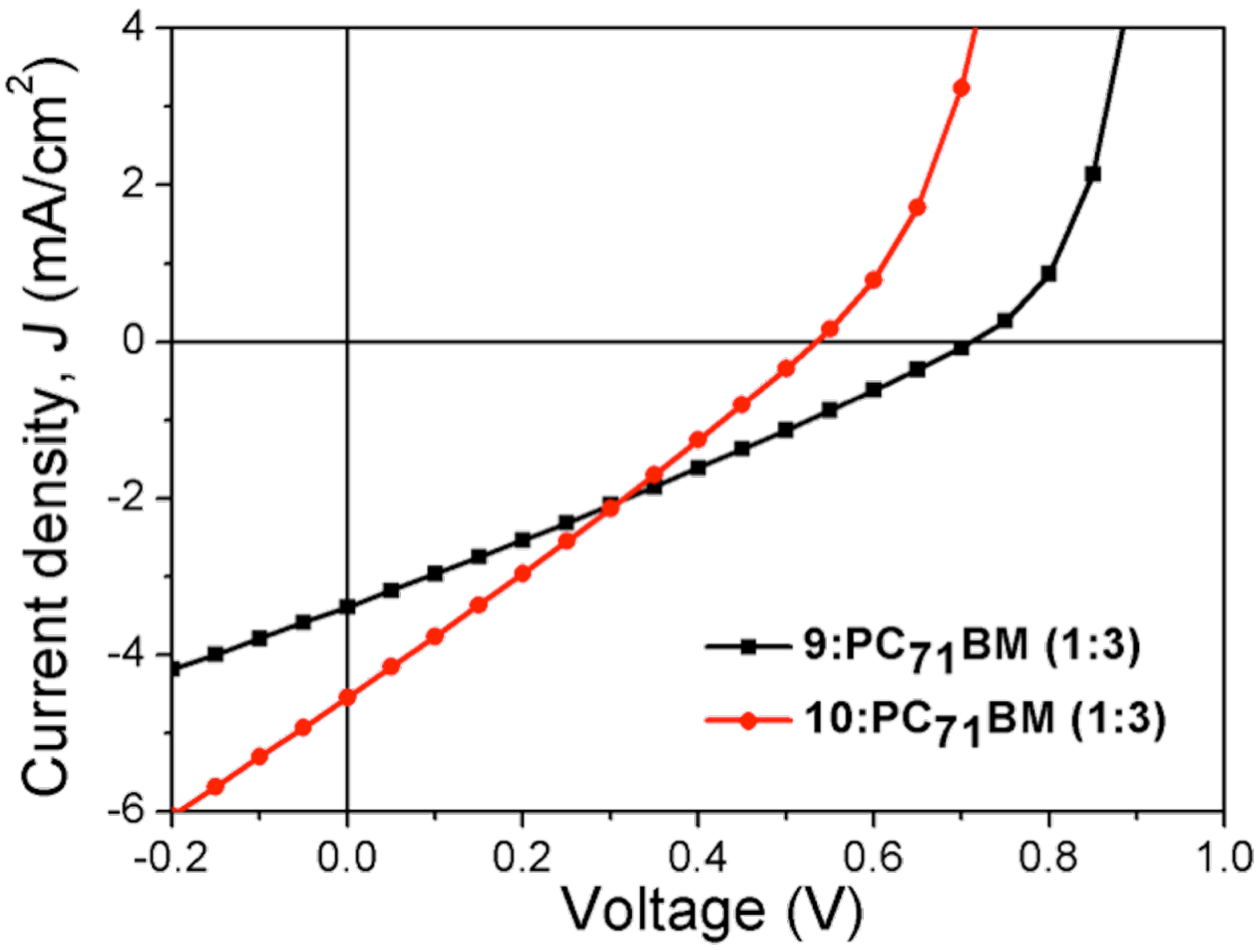
*J*–*V* for **9**:PC_71_BM (1:3) and **10**:PC_71_BM (1:3) under illumination at 100 mW cm^−2^ with an AM1.5 G source.

The dark and illuminated *J*–*V* curves corresponding to these EQE spectra are shown in Figure 10 and Figure 11, respectively. It is important to mention that we noticed an issue concerning the photodegradation of these materials. We have not provided any quantitative analysis of this phenomenon as it is beyond the remit of this study. However, it is noticed when we compare the calculated and measured *J*_sc_. The calculated *J*_sc_ for DPP derivatives is greater than 3 mA cm^−2^.

The performance of compounds **9** and **10** as the active layer in OPV devices are shown in Table 4. We observe a dramatic difference in *V*_oc_ between **9**:PC_71_BM and **10**:PC_71_BM of 0.71 and 0.53 V, respectively. A favourable dark current is observed with **9**:PC_71_BM when compared with **10**:PC_71_BM (Figure 10) for the same donor acceptor concentration ratio. There are several possible reasons why we observe a lower *V*_oc_ with **10**:PC71BM than **9**:PC71BM. Firstly, **10**:PC71BM when compared with **9**:PC71BM has a shallow HOMO and the theoretical maximum *V*_oc_ out of a device is defined as the energy difference between the LUMO of the acceptor and the HOMO of the donor. However, this difference is small. A more compelling reason is that **10**:PC71BM has a propensity to aggregate. As such, an investigation into the morphology of the optimised blends was carried out. Wide-field images indicate more aggregates in the blend **10**:PC_71_BM (1:3) compared to that in **9**:PC_71_BM (1:3). These aggregates are contained within red circles (Figures S9 and S10, Supporting Information File 1).

**Table 4:** Performance of **9**:PC_71_BM (1:3) and **10**:PC_71_BM (1:3) under illumination at 100 mW cm^−2^ with an AM1.5 G source.

Active layer	*J*_sc_ [mA/cm^2^]	*V*_oc_ [V]	FF [%]	PCE [%]

**9**:PC_71_BM (1:3)	3.39	0.71	27	0.65
**10**:PC_71_BM (1:3)	4.55	0.53	26	0.64

**Figure 12:**
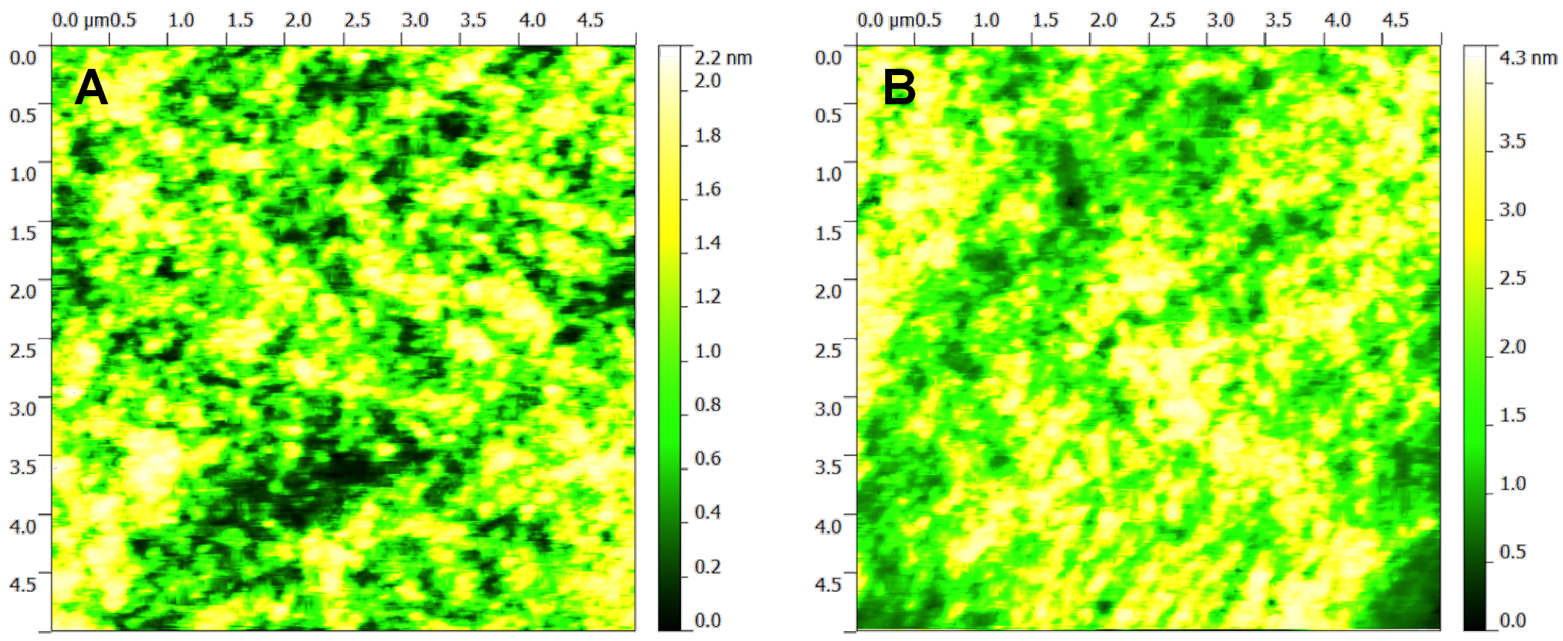
Tapping mode AFM height images for **9**:PC_71_BM (1:3) (left) and **10**:PC_71_BM (1:3) (right) on fused silica.

AFM images (Figure 12) show bead-like aggregates for **9**:PC_71_BM (1:3) and **10**:PC_71_BM (1:3). The aggregates are larger for **10**:PC_71_BM (1:3) than **9**:PC_71_BM (1:3), while the maximum height observed with **10**:PC_71_BM (1:3) is 4.3 nm compared to 2.2 nm for **9**:PC_71_BM (1:3). These small aggregates are interesting, but are not of serious concern. However, the aggregates observed in the wide-field (Figures S9 and S10, Supporting Information File 1) and from the Dektak profiler (Figures S7 and S8, Supporting Information File 1) are of concern for device performance, since the active layer is approximately 70 nm thick (Figure S7, Supporting Information File 1). Figures S11 and S12 (Supporting Information File 1) show tapping mode AFM images of height and phase for **10**:PC_71_BM (1:3). Here we see an aggregate of 5.1 nm height, which is more than double the size of that for **9**:PC_71_BM (1:3). To further investigate aggregation we prepared films of neat **9** and **10** for PLQY measurements. At an excitation of 550 nm we acquired a PLQY of 1.5% for **9** and 0.8% for **10**. The spectra from the integrating sphere are shown in Figures S5 and S6 (Supporting Information File 1). This difference in PLQY is a firm indication of the tendency for **10** to aggregate to a greater degree than **9**. We believe these two DPP cored derivatives to be of interest for photovoltaic applications. Moreover, we are synthesising some new materials which have superior solubility and will not be prone to aggregate on the microscopic scale.

## Conclusion

Undoubtedly, the importance of having highly absorptive cores in the organic compounds is essential in order to increase the light harvesting for organic photovoltaics. However, other parameters such as good alignment of HOMO–LUMO levels with the acceptor material is also vital for efficient photovoltaic devices. Therefore, we have prepared two novel molecular triads bearing the highly absorptive DPP and BODIPY units, which have been used for OPVs. The OPVs demonstrated PCEs greater than 0.65%. Although low when compared to devices fabricated with well-studied solution-processable DPP cored small molecules [24,30,52], these devices do show a comparable photoinduced spectral response. A comparatively low fill factor of 26–27% was observed as a result of poor energy level alignment at the anode side leading to low efficiency. Other parameters governing performance such as short-circuit current density (*J*_sc_), and open-circuit voltage (*V*_oc_) of these devices, are remarkable, considering the size of aggregates observed within the active layer, for example an open-circuit voltage of 0.71 V, which is greater than that reported for OPVs with poly(3-hexylthiophene) [53]. These aggregates, observed by means of a profiler and wide-field microscopy, are detrimental to device performance [54]. Hence, the development of similar compounds with improved solubility and a more favourable morphology would hopefully lead to more efficient OPVs. Finally, we acknowledge the very recent work of Ziessel et al., who have reported solar cells incorporating a hybrid thiophene–benzothiadiazole–thiophene–BODIPY derivative with power conversion efficiencies of ca. 1.25% [55]. Whilst the open circuit voltage of our best device was higher than theirs (0.62 V), in Ziessel’s work the fill factor (35%) and short circuit current density (5.8 mA cm^−2^) were higher.

## Experimental

All the chemicals were purchased from Aldrich and Alfa-Aesar and used without further purification. For reactions under anhydrous conditions, the glassware was dried in an oven at 130 °C. Apart from dry DMF, dry solvents were collected through a PureSolv purification system.

^1^H and ^13^C NMR spectra were recorded at room temperature on a Bruker DRX500 at 500 and 125 MHz or a Bruker Avance 400 instrument at 400 and 100 MHz; chemical shifts are given in ppm and all *J* values are in Hz. MALDI–TOF–MS were recorded on a Shimadzu Axima-CFR spectrometer (mass range 1–150,000 Da). Column chromatography was carried out on VWR silica gel (40–63 µm mesh). Solvents were removed using a rotary evaporator (vacuum supplied by low vacuum pump) and, where necessary, a high vacuum pump was used to remove residual solvent.

Compounds **3** [56], **4** [56], **6** [47], and **7** [47] were prepared according to the literature.

**10,10'-(5',5'''-(2,5-Bis(2-octyldodecyl)-3,6-dioxo-2,3,5,6-tetrahydropyrrolo[3,4-*c*]pyrrole-1,4-diyl)bis([2,2'-bithiophene]-5',5-diyl))bis(2,8-diethyl-5,5-difluoro-1,3,7,9-tetramethyl-5*H*-dipyrrolo[1,2-*c*:2',1'-*f*][1,3,2]diazaborinin-4-ium-5-uide) (9):** DPP **8** (100 mg, 0.09 mmol, 3 equiv), BODIPY **6** (124 mg, 0.3 mmol, 1 equiv), Pd_2_(dba)_3_ (20 mg, 0.02 mmol) and tri-*tert*-butylphosphonium tetrafluoroborate (20 mg, 0.06 mmol) were dissolved in dry THF (10 mL). A solution of tripotassium phosphate (84 mg, 0.4 mmol) in water (3 mL) was added to the previous solution. The reaction was refluxed for 48 hours under nitrogen. Dichloromethane was added to the reaction mixture and washed with water (50 mL), brine (50 mL) and water (50 mL). The organic layer was dried over MgSO_4_, filtered and the solvents evaporated. Column chromatography on silica (eluent mixture, hexane/dichloromethane, 1:1) was carried out. The main fractions were recrystallised by dissolving in dichloromethane and precipitating with methanol. The precipitate was dissolved in hot hexane and the beaker was left in the fridge. The precipitate was filtered and a dark purple solid was obtained (83 mg, 55%). ^1^H NMR (CDCl_3_) 8.88 (d, *J* = 4.1, 2H), 7.37 (m, 4H), 6.96 (d, *J* = 3.6, 2H), 4.05 (d, *J* = 6.8, 4H), 2.55 (s, 12H), 2.35 (m, 8H), 1.98 (br s, 2H), 1.66 (s, 12H), 1.40–1.15 (m, 64H), 1.02 (t, 12H), 0.85 (m, 12H); ^13^C NMR (CDCl_3_) 161.1, 154.4, 141.1, 138.9, 137.9, 137.6, 135.9, 135.8, 132.9, 131.0, 130.1, 128.7, 128.1, 124.7, 124.6, 108.2, 45.8, 37.5, 31.4, 31.3, 30.8, 29.5, 29.1, 29.09, 29.04, 28.8, 28.7, 25.8, 22.1, 16.6, 14.0, 13.5, 12.1, 10.8; MALDI–MS *m*/*z*: 1628.3 [M^+^]; Anal. calcd for C_96_H_134_B_2_F_4_N_6_O_2_S_4_: C, 70.74; H, 8.29; N, 5.16; S, 7.87; found: C, 68.78; H, 8.05; N, 5.56; S, 8.09; MP: 165–167 °C.

**10,10'-(5'',5'''''-(2,5-Bis(2-octyldodecyl)-3,6-dioxo-2,3,5,6-tetrahydropyrrolo[3,4-*c*]pyrrole-1,4-diyl)bis([2,2':5',2''-terthiophene]-5'',5-diyl))bis(2,8-diethyl-5,5-difluoro-1,3,7,9-tetramethyl-5*H*-dipyrrolo[1,2-*c*:2',1'-*f*][1,3,2]diazaborinin-4-ium-5-uide) (10):** BODIPY **7** (278 mg, 0.5 mmol, 2.5 equiv), DPP **8** (218 mg, 0.2 mmol, 1 equiv), Pd_2_(dba)_3_ (40 mg, 0.04 mmol) and tri-*tert*-butylphosphonium tetrafluoroborate (40 mg, 0.1 mmol) were dissolved in dry THF (20 mL). To the previous solution, a solution of tripotassium phosphate (84 mg, 0.4 mmol) in water (3 mL) was added. The reaction was refluxed for 48 hours under nitrogen. Dichloromethane (50 mL) was added to the reaction mixture and washed with water (50 mL), brine (50 mL) and water (50 mL). The organic layer was dried over MgSO_4_, filtered and the solvents evaporated. The resulting solids were loaded onto a silica column (eluent mixture, hexane/dichoromethane, 2:1). The product was subjected to further chromatographic columns in silica (eluent mixture, hexane/ethyl acetate, 7:3). Preparative HPLC (isocratic) was then carried out (eluent mixture, hexane/dichoromethane, 2:1) to obtain **10** as a dark purple solid (120 mg, 34%). ^1^H NMR (CDCl_3_) 8.93 (d, *J* = 4.1, 2H), 7.35 (m, 4H), 7.27–7.24 (2H, (partially masked by CDCl_3_ peak)), 7.19 (d, *J* = 3.8, 2H), 6.93 (d, *J* = 3.6, 2H), 4.06 (d, *J* = 7.5, 4H), 2.56 (s, 12H), 2.35 (m, 8H), 1.98 (br s, 2H), 1.66 (s, 12H), 1.42–1.16 (m, 64H), 1.02 (t, 12H), 0.85 (m, 12H); ^13^C NMR (CDCl_3_) 161.1, 154.2, 141.5, 138.8, 138.1, 138.0, 136.7, 136.1, 134.9, 134.7, 132.8, 131.1, 130.4, 128.5, 127.9, 125.3, 124.6, 124.4, 123.6, 108.1, 45.8, 37.4, 31.4, 30.8, 29.5, 29.1, 29.0, 28.9, 28.8, 25.9, 22.1, 16.6, 14.0, 13.6, 12.1, 10.8; MALDI–MS *m*/*z*: 1794.2 [M^+^]; Anal. calcd for C_104_H_138_B_2_F_4_N_6_O_2_S_6_: C, 69.62; H, 7.75; N, 4.68; S, 10.72; found: C, 67.33; H, 7.60; N, 4.87; S, 11.00; MP: 109–111 °C.

### Device fabrication

Indium tin oxide (ITO) coated glass substrates from Xin Yan Technology Ltd. (15 Ω /□) were masked and etched in hydrochloric acid (37%) for 20 minutes in order to get 4 mm wide strips. The substrates were then cleaned using an ultra sonicator in deionised water, acetone and isopropanol successively. The substrates were then dried with nitrogen and oxygen plasma treated for 3 minutes. Poly(3,4-ethylenedioxythiophene):poly(styrenesulfonate) (PEDOT:PSS) from Clevios (AI4083) was spin-coated at 4000 RPM in order to obtain a 20 nm thin layer on top of the ITO. The PEDOT:PSS coated ITO samples were then placed on a hotplate inside a nitrogen filled glove box (O_2_ < 0.1 PPM, H_2_O < 0.5 PPM) and baked at 120 °C for 20 min in order to remove residual solvents. Films containing various donor (**9** and **10**)/acceptor ratios (1:2, 1:3 and 1:4) were spin-coated from a 20 mg mL^−1^ chlorobenzene solution. [6,6]-Phenyl-C_71_-butyric acid methyl ester (PC_71_BM) from Solenne B. V. Company was used as the acceptor. The devices were then annealed at 140 °C for 20 minutes before being placed into an evaporator for back electrode deposition. 20 nm of calcium and 200 nm of aluminium were thermally evaporated at a base pressure of 2 × 10^−6^ mbar. Devices were then encapsulated with a glass cover slip and a UV curable optical adhesive from Thorlabs. The active area of the devices was 6 mm^2^. Characterisation was performed in air using a Keithley 2400 source-measure unit and a Steuernagel AM 1.5 G solar simulator at 100 mW cm^−2^. The illumination intensity was verified and calibrated with an NREL calibrated mono-silicon detector with KG5 filter. External quantum efficiency (EQE) measurements were performed with an incident photon to charge carrier efficiency (IPCE) setup consisting of a NPL calibrated photodiode, Keithley 6517A picoammeter and a TMc300 monochromator.

For microscopy and photophysical studies, films were prepared from chlorobenzene on fused silica substrates. Neat films were prepared from a 10 mg mL^−1^ solution and composite films from a 20 mg mL^−1^ solution, respectively. Film thicknesses were measured using a Dektak 150 M stylus profiler. Absorption and emission spectra of compounds **9** and **10** were obtained with a Varian Cary 300 UV–visible spectrophotometer and a Photoluminescence Quantum Yield (PLQY) measurement system (model: C9920-02G), respectively. Solution emission spectra were attained for samples dissolved in dichloromethane with a FluoroMax 2 spectrometer. For microscopy a WiTec AlphaSNOM was used for wide-field images and a Veeco scanning probe microscope (SPM) was used in tapping mode for atomic force microscopy (AFM).

### Theoretical calculations

DFT optimisations were carried out in TURBOMOLE 6.3.1 [57] using B97-D [58] functional with def2-TZVP [59] basis set in dichloromethane using COSMO [60] solvent model. RI-J [61] approximation was implemented for these optimisations. TDDFT [62] and CHelpG [51] calculations were performed using wB97XD [63] functional with TZVP [64] basis set and SMD [65] solvent model implemented in Gaussian 09 [66]. Alkyl chains were shortened to methyl groups to lessen the computational cost.

## Supporting Information

File 1 Absorption and emission spectra of compounds **9** and **10** and their fullerene blends; film thickness measurements; surface analysis; representation of device structure; device characteristics; computational data.

## References

[1] Sariciftci N S, Smilowitz L, Heeger A J, Wudl F (1992). Science.

[2] Yu G, Gao J, Hummelen J C, Wudl F, Heeger A J (1995). Science.

[3] Halls J J M, Walsh C A, Greenham N C, Marseglia E A, Friend R H, Moratti S C, Holmes A B (1995). Nature.

[4] Ma W, Yang C, Gong X, Lee K, Heeger A J (2005). Adv Funct Mater.

[5] Hummelen J C, Knight B W, LePeq F, Wudl F, Yao J, Wilkins C L (1995). J Org Chem.

[6] Li Y (2012). Acc Chem Res.

[7] Li G, Zhu R, Yang Y (2012). Nat Photonics.

[8] Cheng Y-J, Yang S-H, Hsu C-S (2009). Chem Rev.

[9] Beaujuge P M, Fréchet J M J (2011). J Am Chem Soc.

[10] Roncali J (2009). Acc Chem Res.

[11] Mishra A, Bäuerle P (2012). Angew Chem, Int Ed.

[12] Lin Y, Li Y, Zhan X (2012). Chem Soc Rev.

[13] Li Y, Guo Q, Li Z, Pei J, Tian W (2010). Energy Environ Sci.

[14] Walker B, Kim C, Nguyen T-Q (2010). Chem Mater.

[15] Peumans P, Yakimov A, Forrest S R (2003). J Appl Phys.

[16] Zhang F, Wu D, Xu Y, Feng X (2011). J Mater Chem.

[17] Wright I A, Kanibolotsky A L, Cameron J, Tuttle T, Skabara P J, Coles S J, Howells C T, Thomson S A J, Gambino S, Samuel I D W (2012). Angew Chem, Int Ed.

[18] Sun Y, Welch G C, Leong W L, Takacs C J, Bazan G C, Heeger A J (2012). Nat Mater.

[19] Wang D H, Kyaw A K K, Gupta V, Bazan G C, Heeger A J (2013). Adv Energy Mater.

[20] Roquet S, Cravino A, Leriche P, Alévêque O, Frère P, Roncali J (2006). J Am Chem Soc.

[21] Roquet S, de Bettignies R, Leriche P, Cravino A, Roncali J (2006). J Mater Chem.

[22] Fitzner R, Mena-Osteritz E, Mishra A, Schulz G, Reinold E, Weil M, Körner C, Ziehlke H, Elschner C, Leo K (2012). J Am Chem Soc.

[23] Haid S, Mishra A, Weil M, Uhrich C, Pfeiffer M, Bäuerle P (2012). Adv Funct Mater.

[24] Qu S, Tian H (2012). Chem Commun.

[25] Nielsen C B, Turbiez M, McCulloch I (2013). Adv Mater.

[26] Tamayo A B, Dang X-D, Walker B, Seo J, Kent T, Nguyen T-Q (2009). Appl Phys Lett.

[27] Tamayo A B, Tantiwiwat M, Walker B, Nguyen T-Q (2008). J Phys Chem C.

[28] Kim C, Liu J, Lin J, Tamayo A B, Walker B, Wu G, Nguyen T-Q (2012). Chem Mater.

[29] Walker B, Tamayo A B, Dang X-D, Zalar P, Seo J H, Garcia A, Tantiwiwat M, Nguyen T-Q (2009). Adv Funct Mater.

[30] Sonar P, Ng G-M, Lin T T, Dodabalapur A, Chen Z-K (2010). J Mater Chem.

[31] Poe A M, Della Pelle A M, Subrahmanyam A V, White W, Wantz G, Thayumanavan S (2014). Chem Commun.

[32] Ulrich G, Ziessel R, Harriman A (2008). Angew Chem, Int Ed.

[33] Boens N, Leen V, Dehaen W (2012). Chem Soc Rev.

[34] Loudet A, Burgess K (2007). Chem Rev.

[35] Alemdaroglu F E, Alexander S C, Ji D, Prusty D K, Börsch M, Herrmann A (2009). Macromolecules.

[36] Popere B C, Della Pelle A M, Thayumanavan S (2011). Macromolecules.

[37] Algı F, Cihaner A (2009). Org Electron.

[38] Kim B, Ma B, Donuru V R, Liu H, Fréchet J M J (2010). Chem Commun.

[39] Cortizo-Lacalle D, Howells C T, Gambino S, Vilela F, Vobecka Z, Findlay N J, Inigo A R, Thomson S A J, Skabara P J, Samuel I D W (2012). J Mater Chem.

[40] Rousseau T, Cravino A, Bura T, Ulrich G, Ziessel R, Roncali J (2009). J Mater Chem.

[41] Rousseau T, Cravino A, Bura T, Ulrich G, Ziessel R, Roncali J (2009). Chem Commun.

[42] Rousseau T, Cravino A, Ripaud E, Leriche P, Rihn S, De Nicola A, Ziessel R, Roncali J (2010). Chem Commun.

[43] Bura T, Leclerc N, Fall S, Lévêque P, Heiser T, Retailleau P, Rihn S, Mirloup A, Ziessel R (2012). J Am Chem Soc.

[44] Hablot D, Retailleau P, Ziessel R (2010). Chem – Eur J.

[45] Hablot D, Harriman A, Ziessel R (2011). Angew Chem, Int Ed.

[46] Hablot D, Sutter A, Retailleau P, Ziessel R (2012). Chem – Eur J.

[47] Benniston A C, Copley G, Harriman A, Ryan R (2011). J Mater Chem.

[48] Cortizo-Lacalle D, Arumugam S, Elmasly S E T, Kanibolotsky A L, Findlay N J, Inigo A R, Skabara P J (2012). J Mater Chem.

[49] Roncali J (1997). Chem Rev.

[50] Benniston A C, Copley G, Harriman A, Rewinska D B, Harrington R W, Clegg W (2008). J Am Chem Soc.

[51] Breneman C M, Wiberg K B (1990). J Comput Chem.

[52] Tamayo A B, Dang X-D, Walker B, Seo J, Kent T, Nguyen T-Q (2009). Appl Phys Lett.

[53] Ameri T, Min J, Li N, Machui F, Baran D, Forster M, Schottler K J, Dolfen D, Scherf U, Brabec C J (2012). Adv Energy Mater.

[54] Arumugam S, Wright I A, Inigo A R, Gambino S, Howells C T, Kanibolotsky A L, Skabara P J, Samuel I D W (2014). J Mater Chem C.

[55] Mirloup A, Leclerc N, Rihn S, Bura T, Bechara R, Hébraud A, Lévêque P, Heiser T, Ziessel R (2014). New J Chem.

[56] Wu Z, An Z, Chen X, Chen P (2013). Org Lett.

[57] Ahlrichs R, Bär M, Häser M, Horn H, Kölmel C (1989). Chem Phys Lett.

[58] Grimme S (2006). J Comput Chem.

[59] Weigend F, Ahlrichs R (2005). Phys Chem Chem Phys.

[60] Klamt A, Schüürmann G (1993). J Chem Soc, Perkin Trans 2.

[61] Feyereisen M, Fitzgerald G, Komornicki A (1993). Chem Phys Lett.

[62] Runge E, Gross E K U (1984). Phys Rev Lett.

[63] Chai J-D, Head-Gordon M (2008). Phys Chem Chem Phys.

[64] Schäfer A, Huber C, Ahlrichs R (1994). J Chem Phys.

[65] Marenich A V, Cramer C J, Truhlar D G (2009). J Phys Chem B.

[66] (2009). Gaussian 09.

