# Supplementary material for: Solution processable diketopyrrolopyrrole (DPP) cored small molecules with BODIPY end groups as novel donors for organic solar cells
Source: Beilstein J Org Chem. 2014 Nov 18;10:2683–95. doi: 10.3762/bjoc.10.283 (PMC4273300; doi:10.3762/bjoc.10.283)
Supplement: File 1 — Absorption and emission spectra of compounds 9 and 10 and their fullerene blends; film thickness measurements; surface analysis; representation of device structure; device characteristics; computational data. [file Beilstein_J_Org_Chem-10-2683-s001.pdf]

Supporting Information

for

Solution processable diketopyrrolopyrrole (DPP) cored small molecules with BODIPY end groups as novel donors for organic solar cells

Diego Cortizo-Lacalle[1], Calvyn T. Howells[2], Upendra K. Pandey[2,3], Joseph Cameron[1], Neil J. Findlay[1], Anto Regis Inigo[1], Tell Tuttle[1], Peter J. Skabara[*1] and Ifor D. W. Samuel[*2]

Address: [1]WestCHEM, Department of Pure and Applied Chemistry, University of Strathclyde, Glasgow, G1 1XL, UK; [2]Organic Semiconductor Centre, SUPA, School of Physics & Astronomy, University of St. Andrews, St. Andrews, KY16 9SS, UK and [3]Present address: Interdisciplinary Centre for Energy Research, Indian Institute of Science, Bangalore 560012, India

Email: Peter J. Skabara[*] - peter.skabara@strath.ac.uk; Ifor D. W. Samuel - idws@st-andrews.ac.uk

*Corresponding author

Absorption and emission spectra of compounds 9 and 10 and their fullerene blends; film thickness measurements; surface analysis; representation of device structure; device characteristics; computational data.

Photophysics

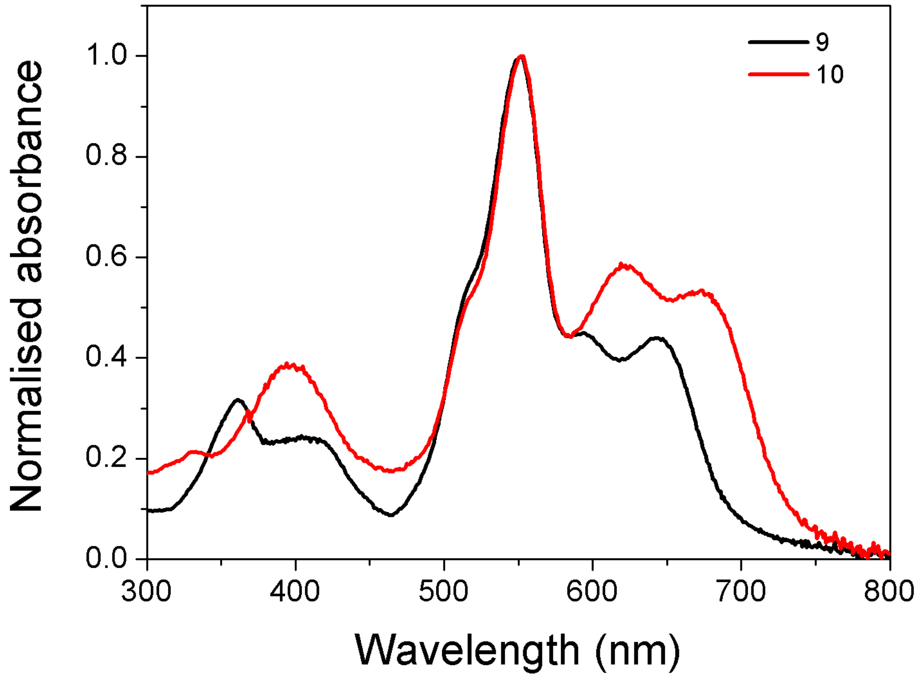

Figure S1: Solid state absorption spectra of **9** (black) and **10** (red), spin-coated from chlorobenzene.

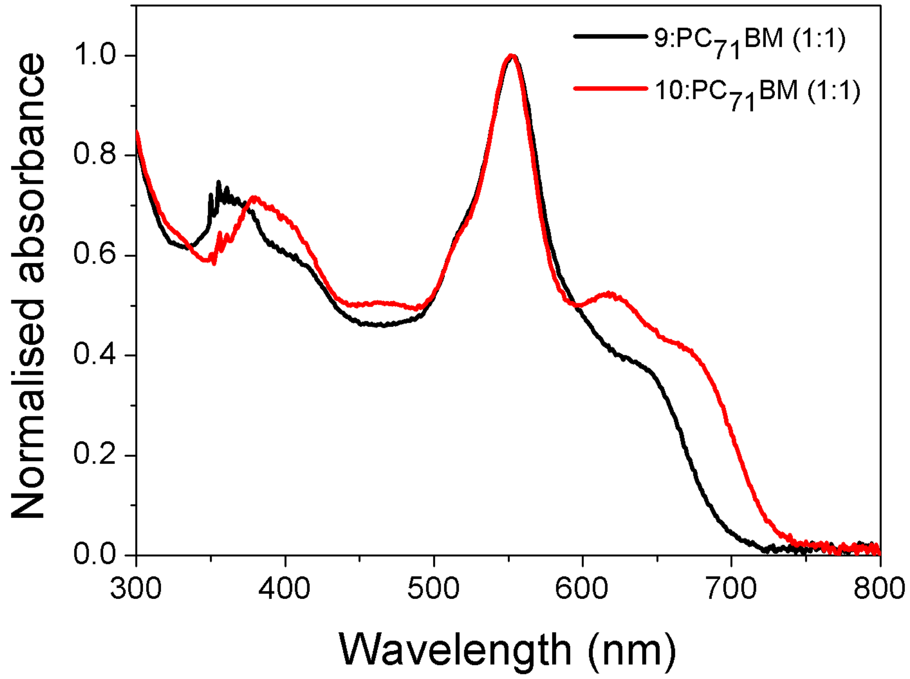

Figure S2: Solid state absorption spectra for **9**:$PC_{71}BM$ (1:1) (black) and **10**:$PC_{71}BM$ (1:1) (red).

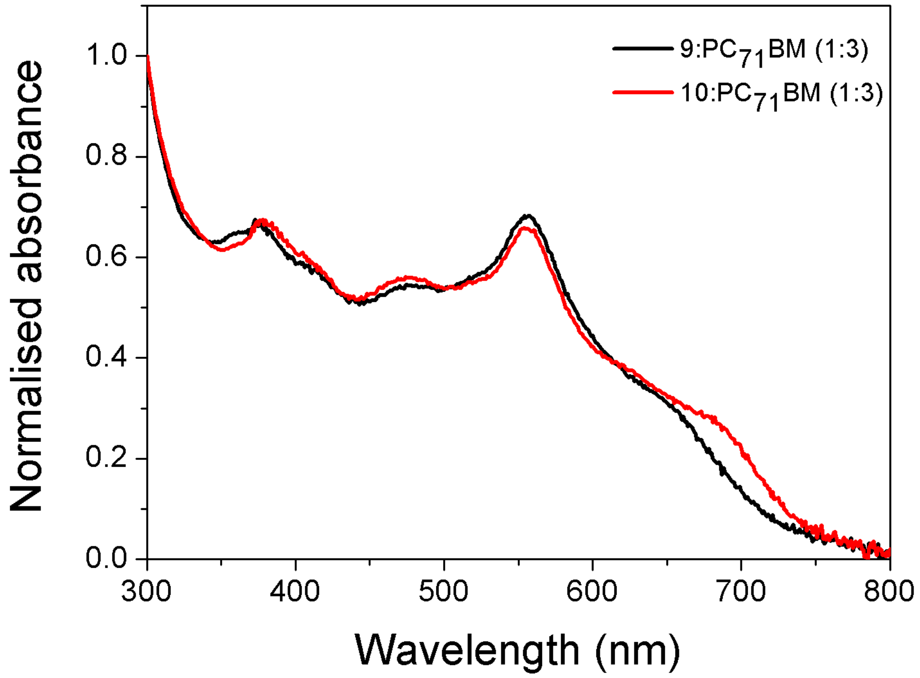

Figure S3: Solid state absorption spectra for **9**:$PC_{71}BM$ (1:3) (black) and **10**:$PC_{71}BM$ (1:3) (red).

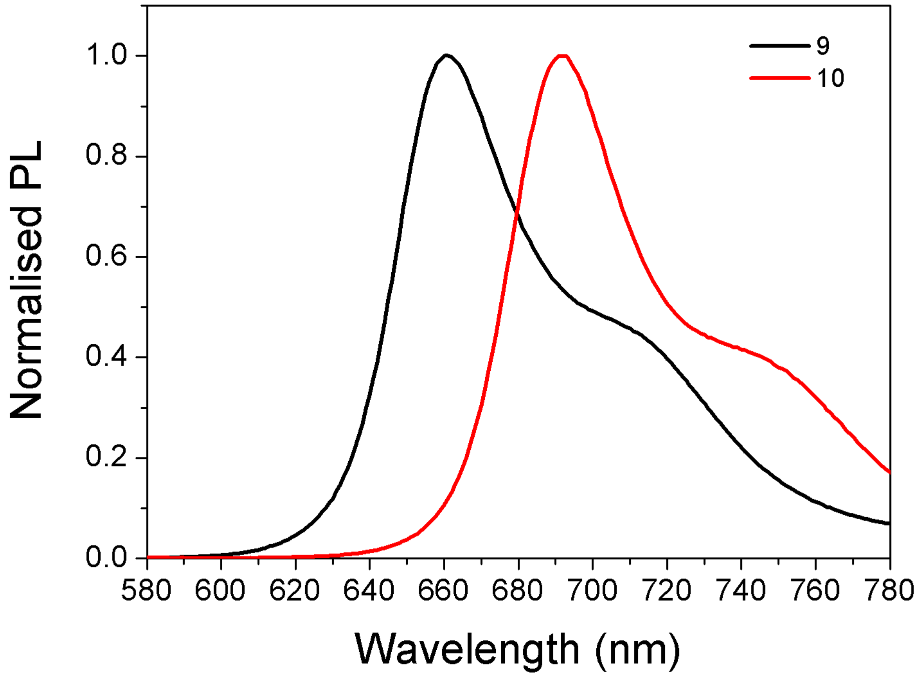

Figure S4: Emission spectra of **9** (black) and **10** (red) acquired from 400 nm excitation (dichlorobenzene solution).

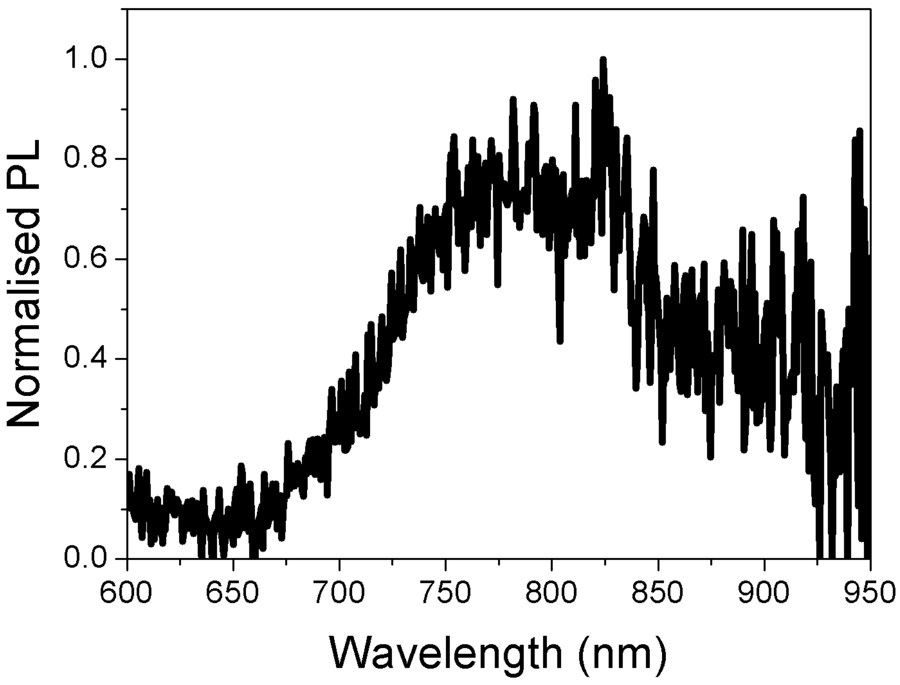

Figure S5: Emission spectrum of **9** acquired from 550 nm excitation (solid state).

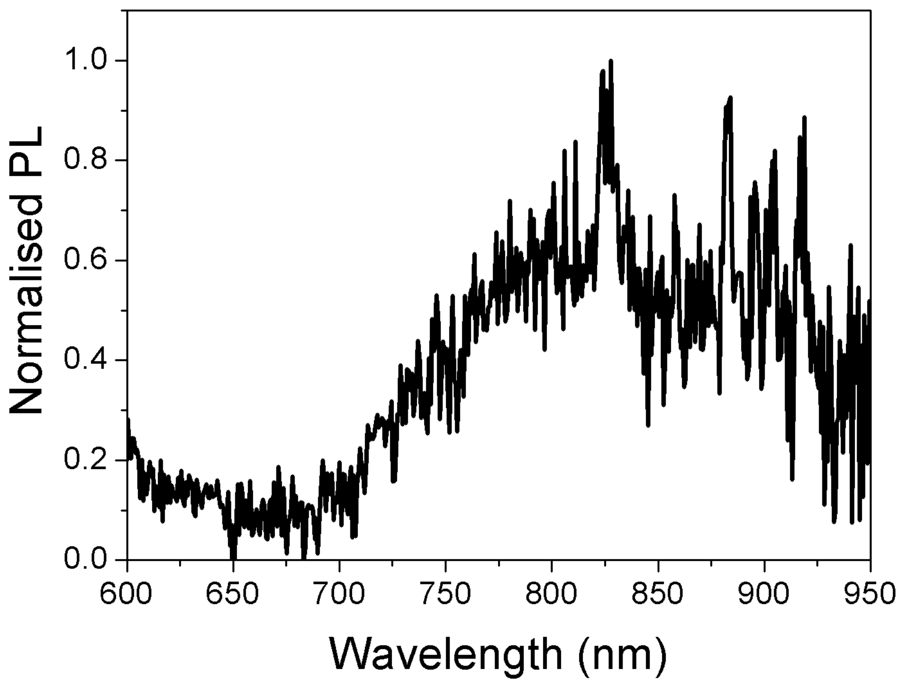

Figure S6: Emission spectrum of **10** acquired from 550 nm excitation (solid state).

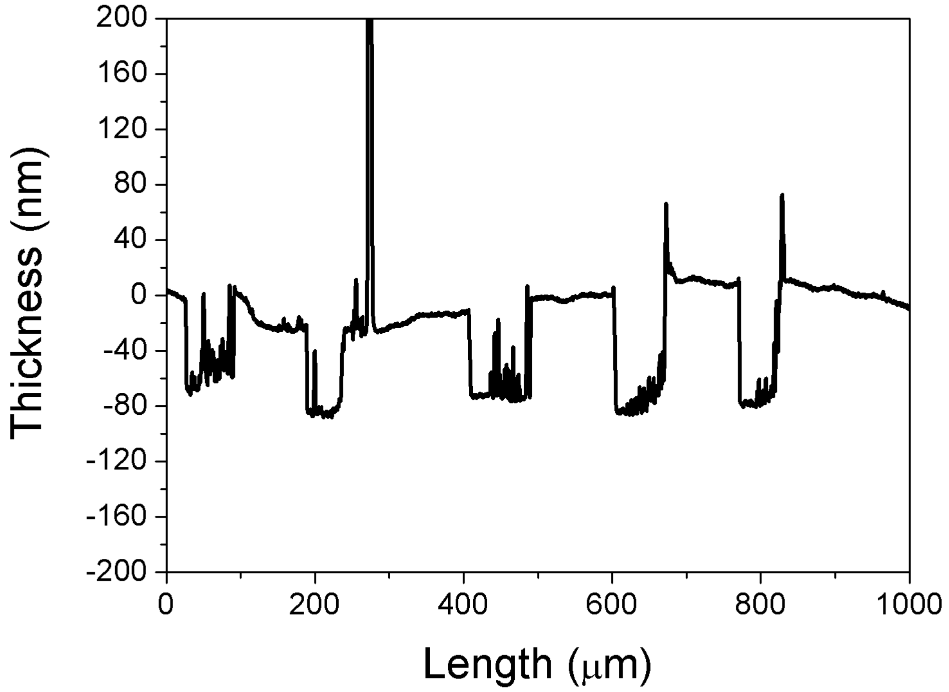

Figure S7: Film thickness of **9**:$PC_{71}BM$ (1:3) from a Dektak profiler. To get an indication of the film thickness the film has been removed from five separate regions.

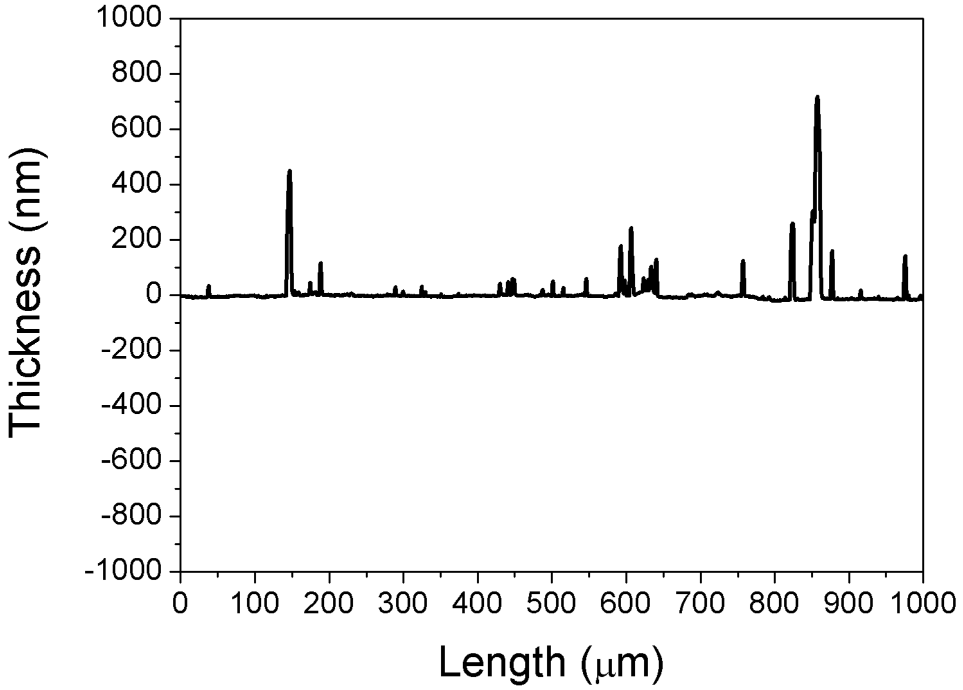

Figure S8: Film thickness of **10**:$PC_{71}BM$ (1:3) from a Dektak profiler.

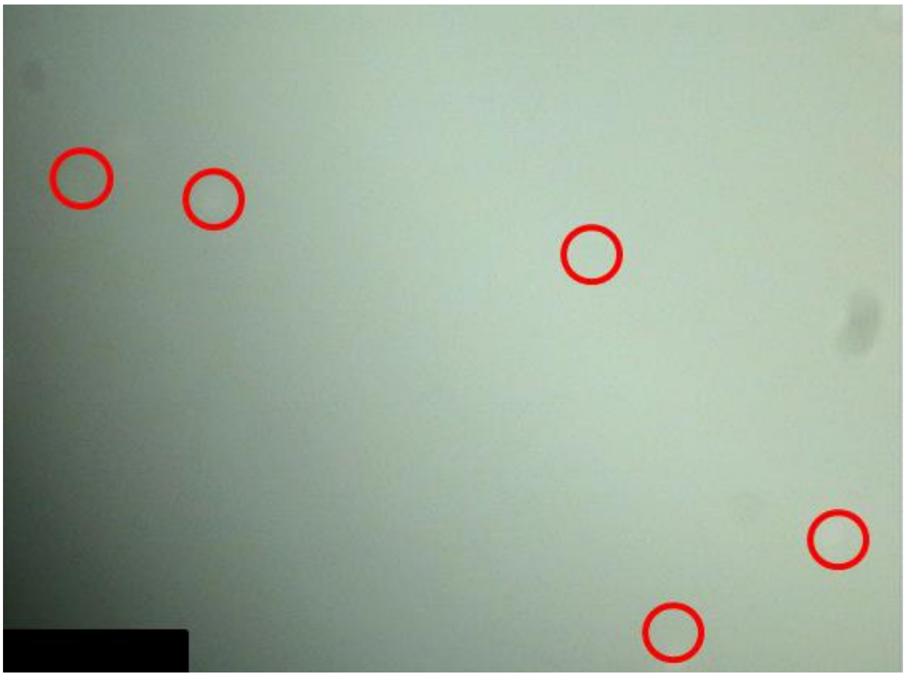

Figure S9: Wide-field of **9**:$PC_{71}BM$ (1:3). The black scale bar in the bottom left hand corner of the image is 80 µm long. In this image there are five regions where aggregates are observed. These regions are defined with red circles.

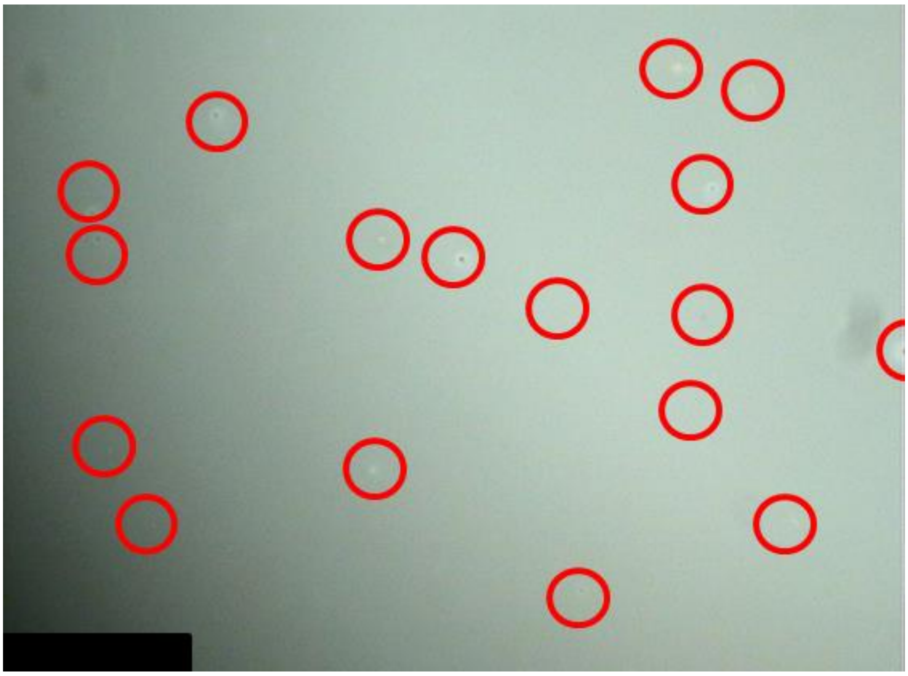

Figure S10: Wide-field of **10**:$PC_{71}BM$ (1:3). The black scale bar in the bottom left hand corner of the image is 80 µm long. In this image there are seventeen regions where aggregates are observed. These regions are defined with red circles.

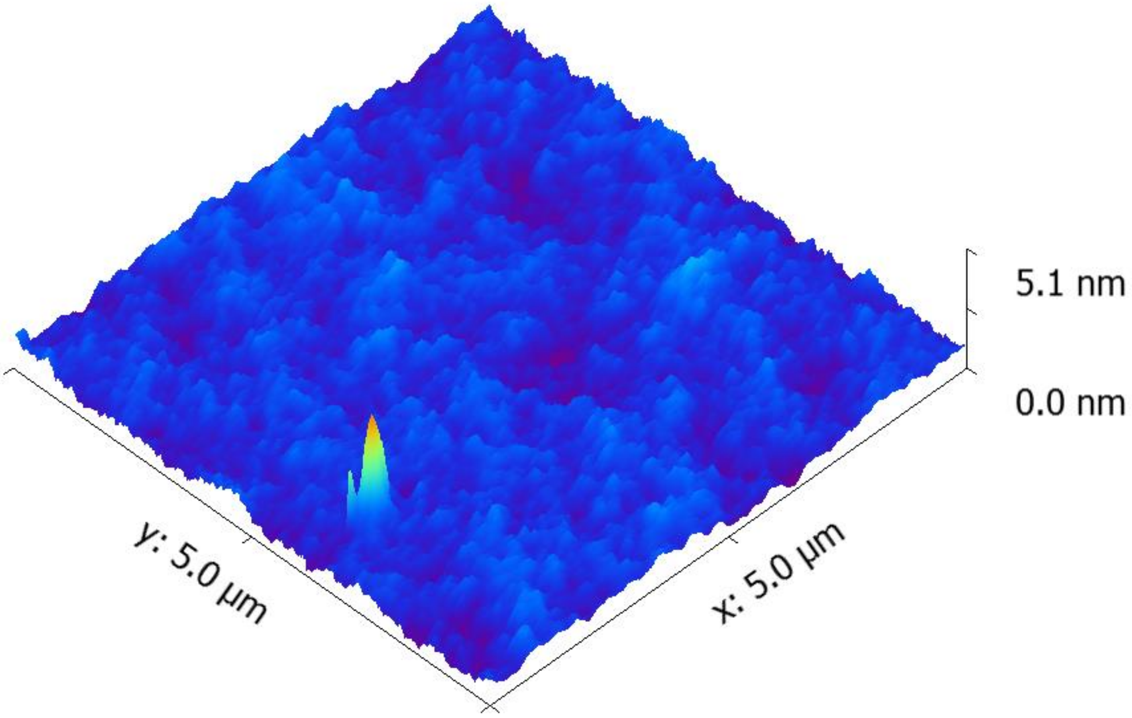

Figure S11: Tapping mode AFM height image of **10**:$PC_{71}BM$ (1:3) on fused silica substrate.

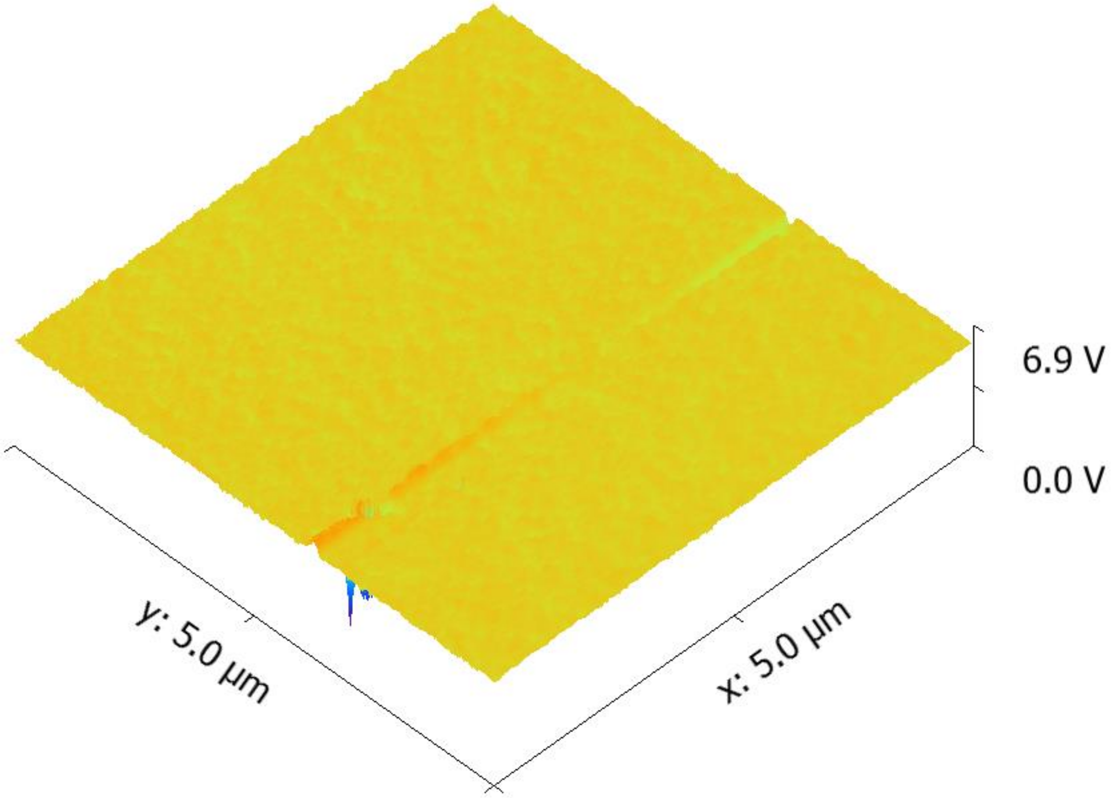

Figure S12: Tapping mode AFM phase image of **10**:$PC_{71}BM$ (1:3) on fused silica substrate.

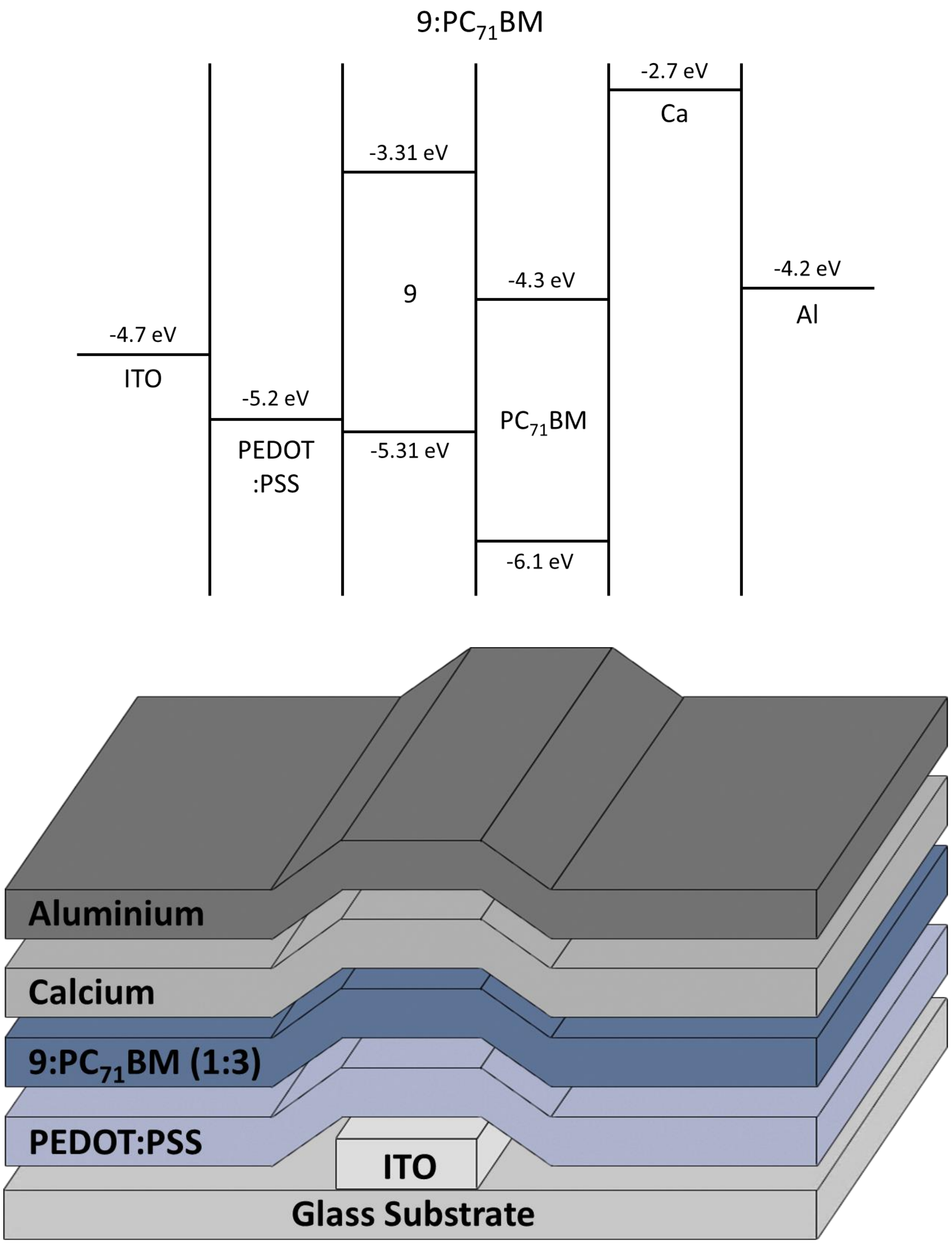

Figure S13: Energy levels (top) and device structure (bottom) for **9**:$PC_{71}BM$ (1:3).

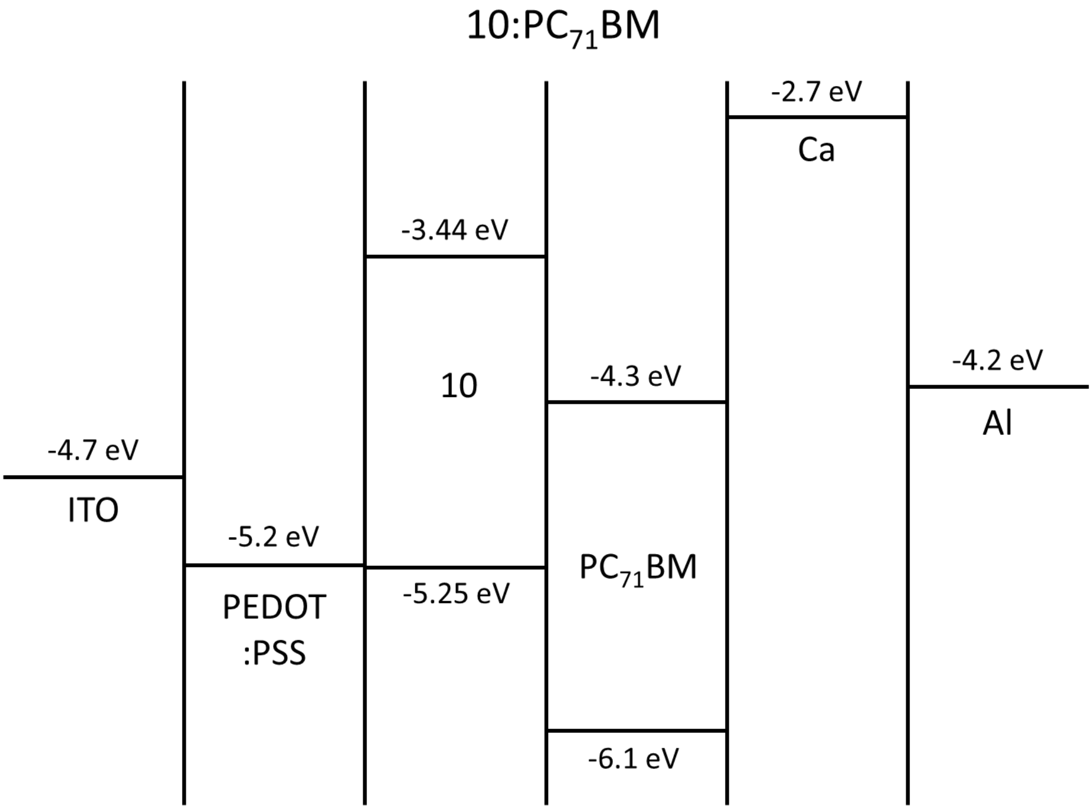

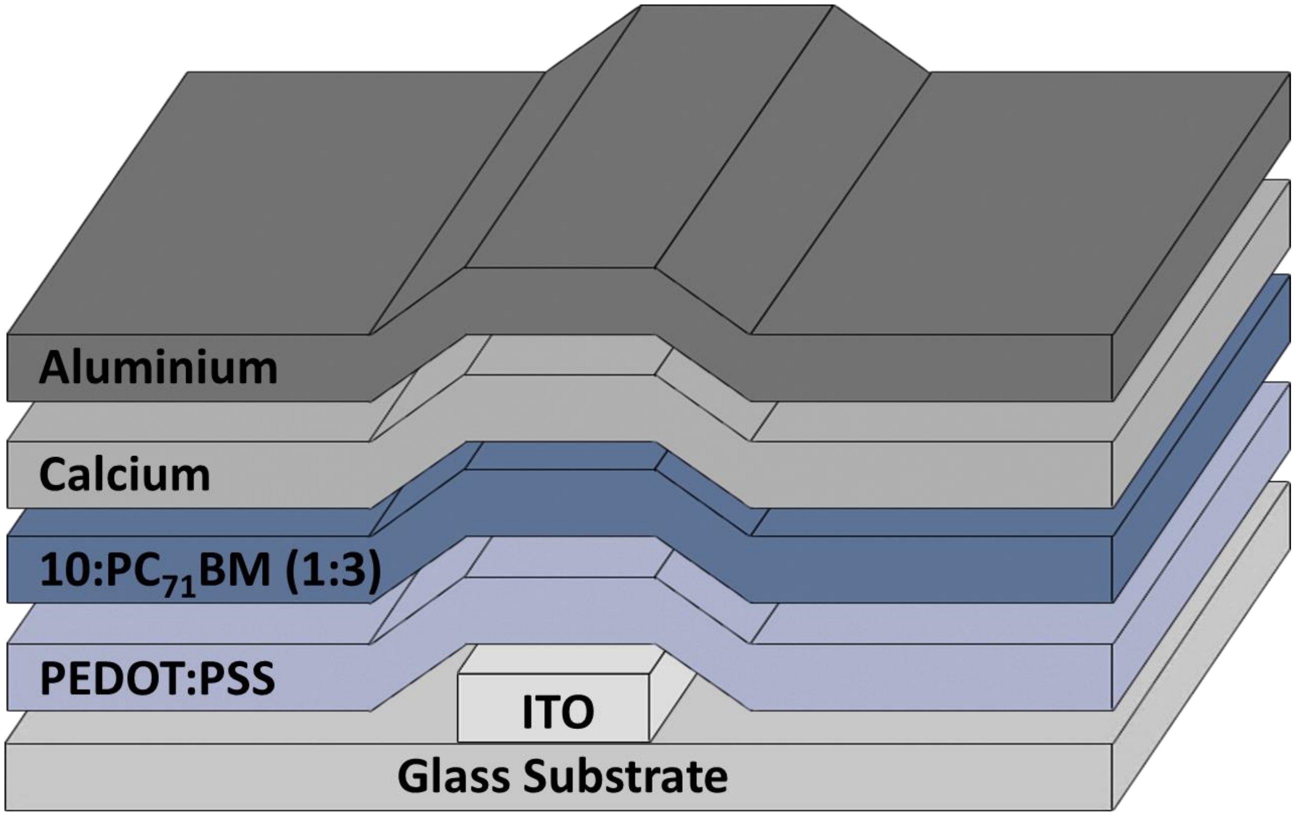

Figure S14: Energy levels (top) and device structure (bottom) for **10**:$PC_{71}BM$ (1:3).

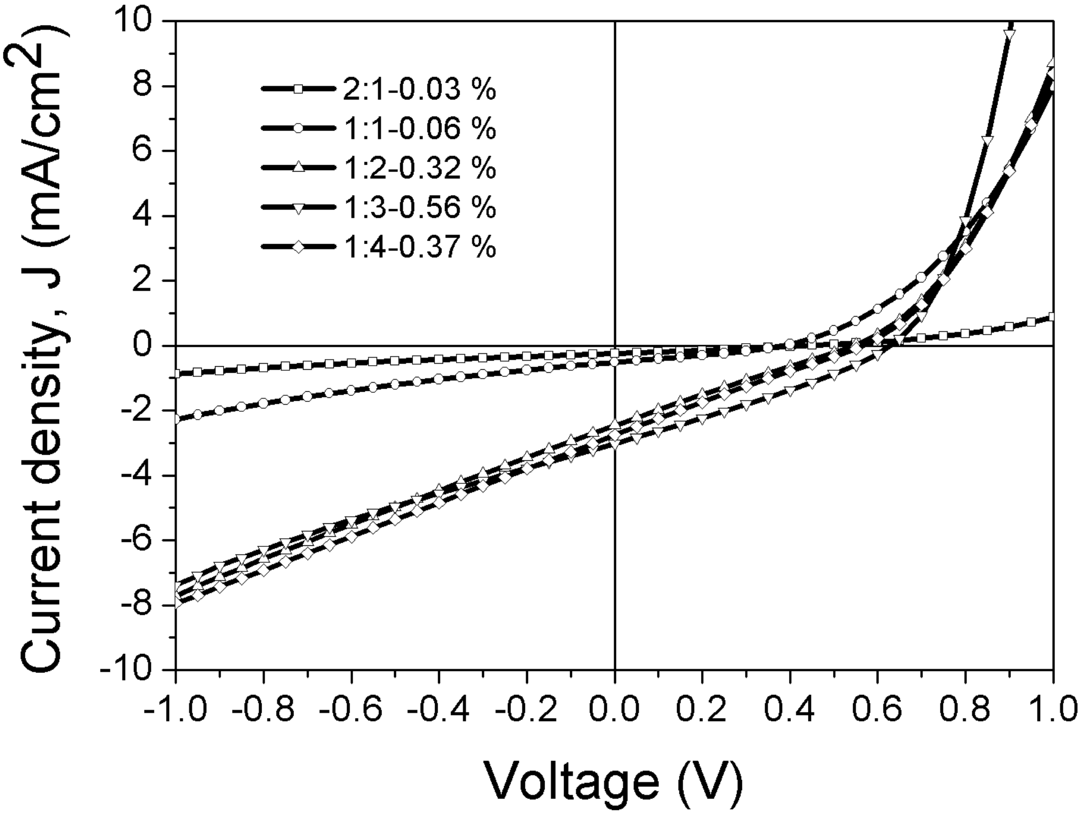

Figure S15: *J*–*V* characteristics of **9**:$PC_{71}BM$ varying donor/acceptor ratios under 100 mW cm^{-2} illumination with a standard AM1.5 G source.

Donor-Acceptor Ratio	Jsc [mA/cm^2]	Voc [V]	FF [%]	PCE [%]
**2:1**	0.24	0.44	28	0.03
**1:1**	0.51	0.39	30	0.06
**1:2**	2.46	0.56	23	0.32
**1:3**	3.02	0.62	30	0.56
**1:4**	2.76	0.57	24	0.37

Figure S16: Device characteristics for various donor/acceptor ratios of **9**:$PC_{71}BM$.

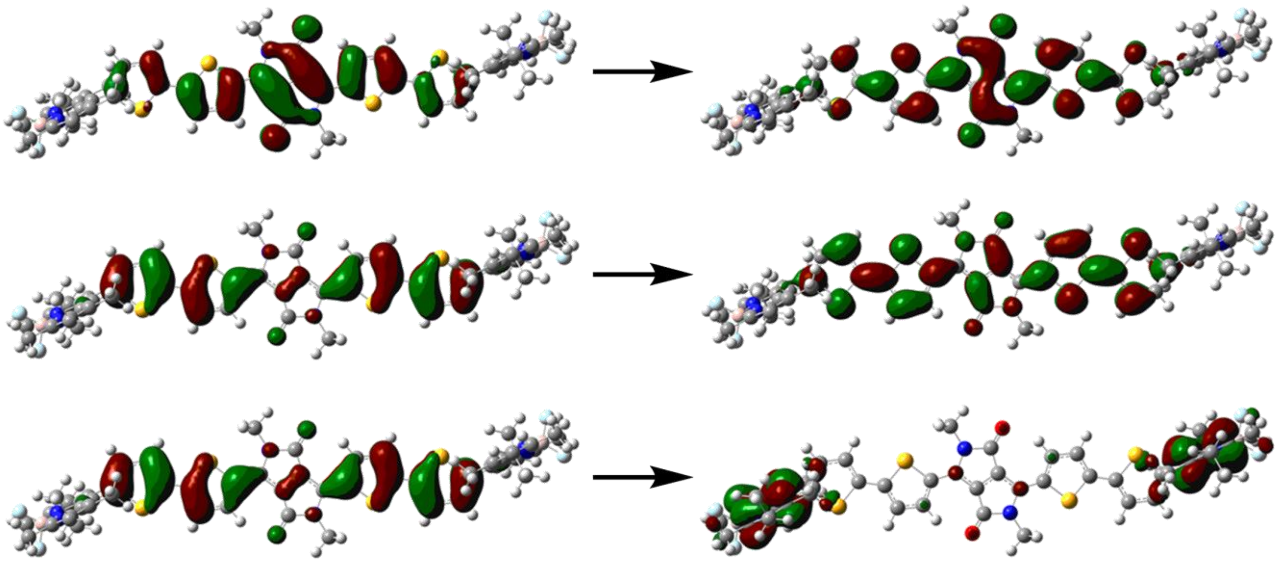

Figure S17: Dominant transitions in TDDFT calculation of **9** at 612 nm.

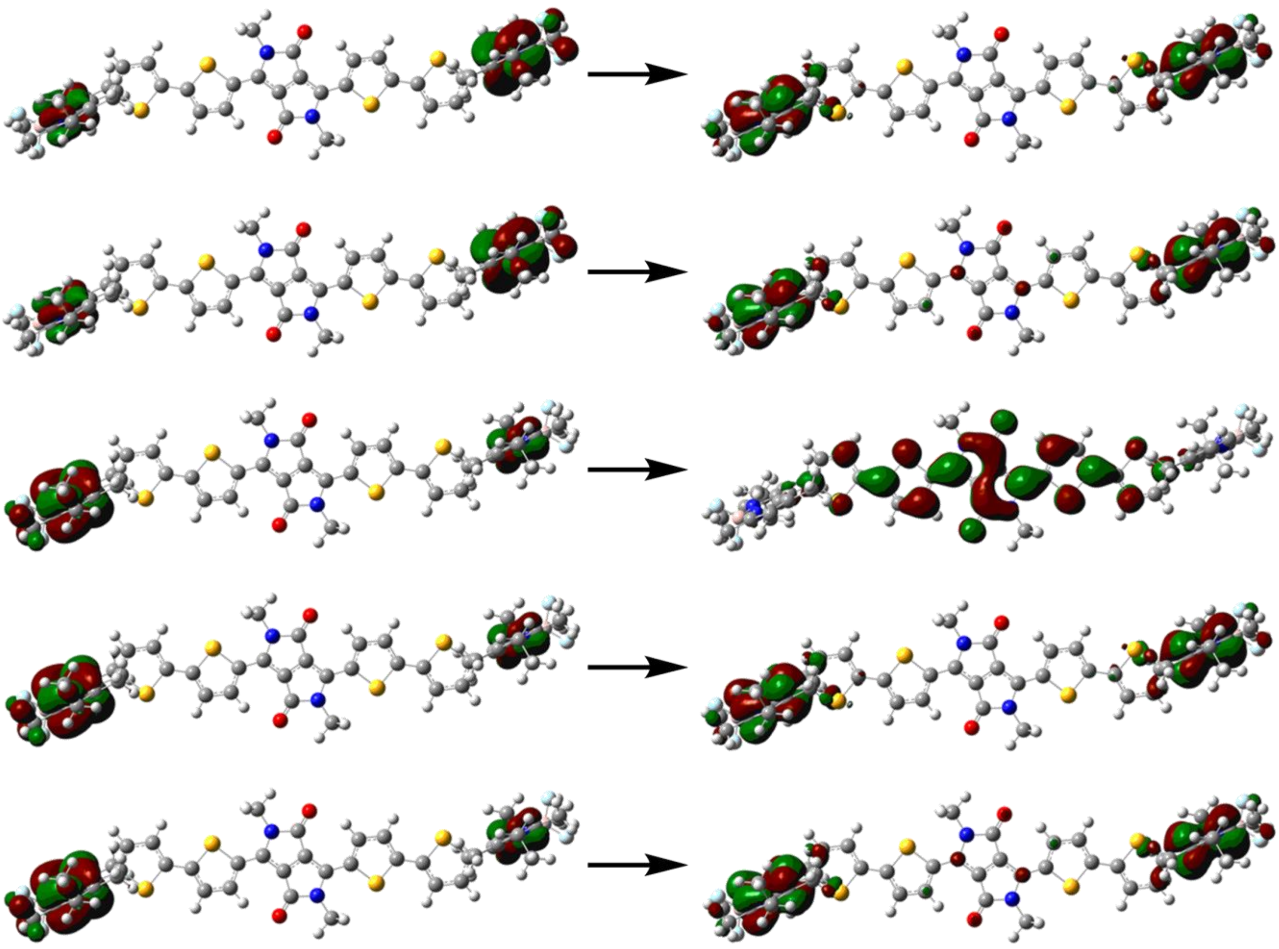

Figure S18: Dominant transitions in TDDFT calculation of **9** at 510 nm.

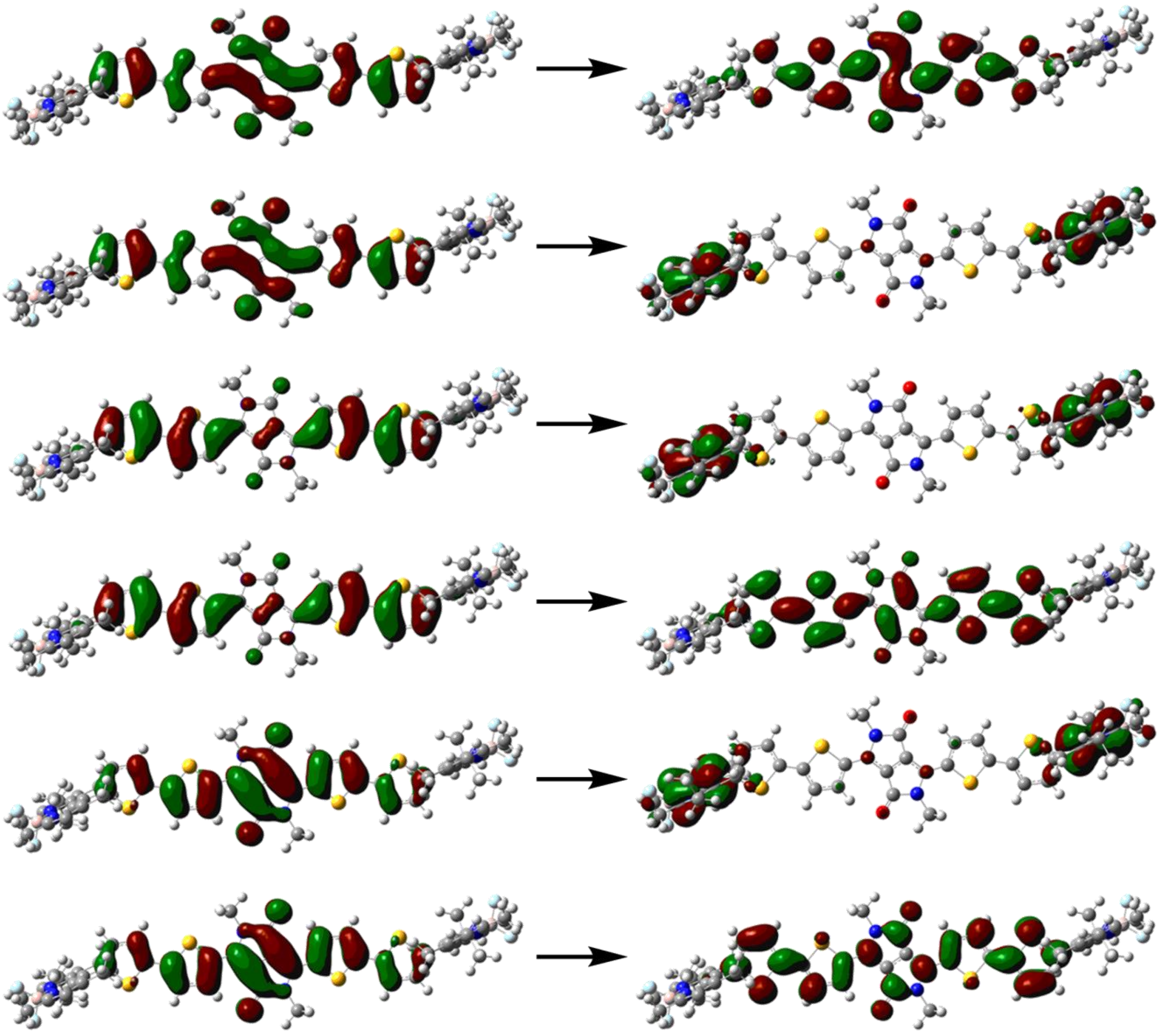

Figure S19: Dominant transitions in TDDFT calculation of **9** at 370 nm.

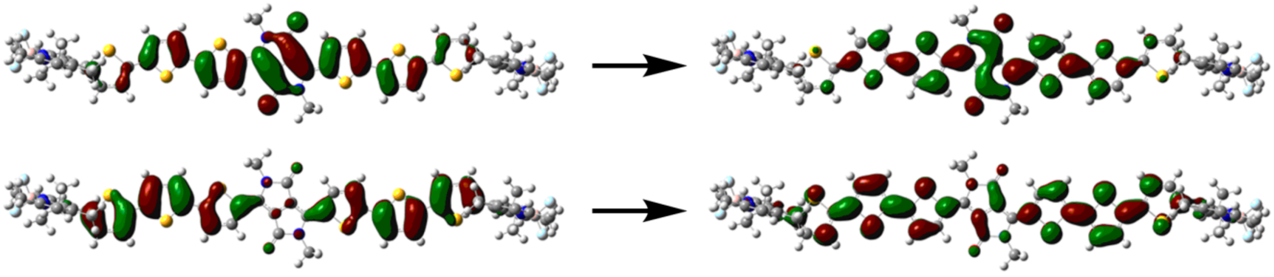

Figure S20: Dominant transitions in TDDFT calculation of **10** at 641 nm.

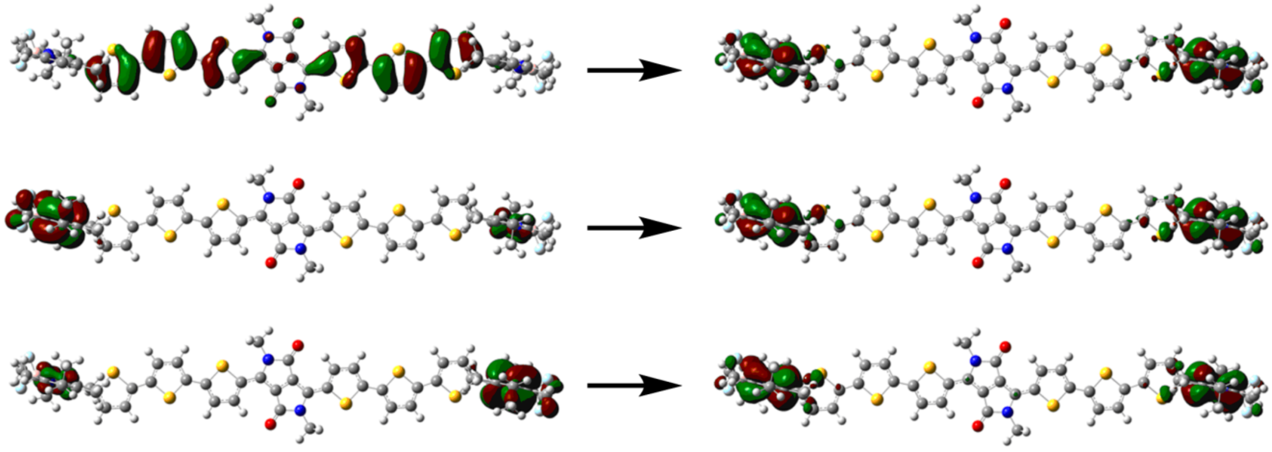

Figure S21: Dominant transitions in TDDFT calculation of **10** at 510 nm.

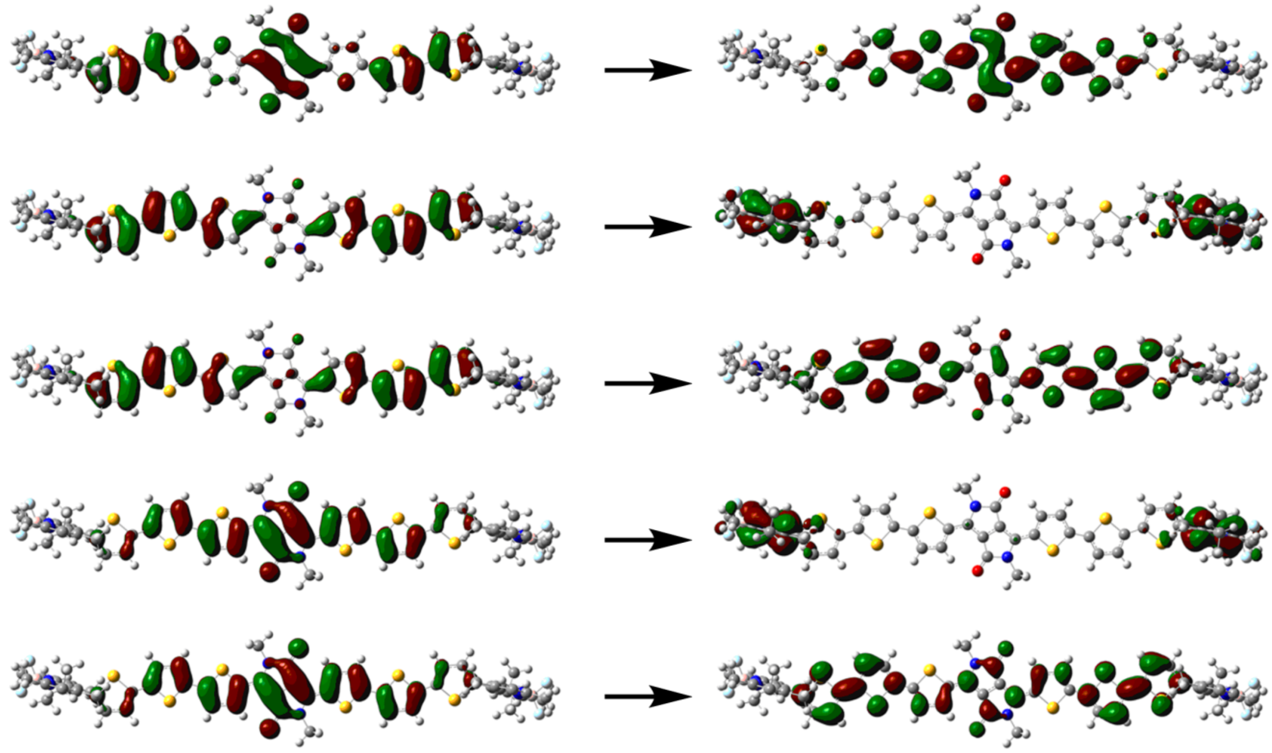

Figure S22: Dominant transitions in TDDFT calculation of **10** at 403 nm.

Table S1. TDDFT Results

Compound	Calculated Absorption peaks / nm	Transitions
**9**	612	HOMO→LUMO **(75%)**; HOMO-3→LUMO+2 **(12%)**; HOMO-3→LUMO+3 **(13%)**
	510	HOMO-2→LUMO+1 **(32%)**; HOMO-2→LUMO+2 **(12%)**; HOMO-1→LUMO **(9%)**; HOMO-1→LUMO+1 **(15%)**; HOMO-1→LUMO+2 **(32%)**
	370	HOMO-4→LUMO **(20%)**; HOMO-4→LUMO+2 **(13%)**; HOMO-3→LUMO+1 **(24%)**; HOMO-3→LUMO+3 **(7%)**; HOMO→LUMO+2 **(27%)**; HOMO→LUMO+4 **(9%)**
**10**	641	HOMO→LUMO **(83%)**; HOMO-3→LUMO+3 **(17%)**
	510	HOMO-3→LUMO+1 **(35%)**; HOMO-2→LUMO+1 **(23%)**; HOMO-1→LUMO+2 **(42%)**
	403	HOMO-4→LUMO **(32%)**; HOMO-3→LUMO+1 **(14%)**; HOMO-3→LUMO+3 **(23%)**; HOMO→LUMO+2 **(10%)**; HOMO→ LUMO+4 **(21%)**
